# Metatranscriptomics of microbial biofilm succession on HDPE foil: uncovering plastic-degrading potential in soil communities

**DOI:** 10.1186/s40793-024-00621-1

**Published:** 2024-11-21

**Authors:** Joana MacLean, Alexander Bartholomäus, Roberts Blukis, Susanne Liebner, Dirk Wagner

**Affiliations:** 1 GFZ German Research Centre for Geosciences, Section Geomicrobiology, 14473 Potsdam, Germany; 2 GFZ German Research Centre for Geosciences, Section Interface Geochemistry, 14473 Potsdam, Germany; 3 Leibniz-Institut für Kristallzüchtung, Max-Born-Str. 2, 12489 Berlin, Germany; 4 Institute of Biochemistry and Biology, University of Potsdam, 14476 Potsdam, Germany; 5 Institute of Geosciences, University of Potsdam, 14476 Potsdam, Germany

**Keywords:** Plastisphere, Soil, Metatranscriptomic, Biofilm, Plastic degradation, Microorganisms

## Abstract

**Background:**

Although plastic pollution is increasing worldwide, very little is known about the microbial processes that take place once plastic debris is incorporated into the soil matrix. In this study, we conducted the first metatranscriptome analysis of polyethylene (PE)-associated biofilm communities in highly polluted landfill soil and compared their gene expression to that of a forest soil community within a 53-day period.

**Results:**

Our findings indicate that the microbial population present in soil contaminated with plastic debris is predisposed to both inhabit and degrade plastic surfaces. Surprisingly, the microbial community from undisturbed forest soil contained a diverse array of plastic-associated genes (*PETase*, *alkB*, etc.), indicating the presence of an enzymatic machinery capable of plastic degradation. Plastic-degrading taxa were upregulated in the early stages of biofilm formation. During the maturation of the biofilm, the *alkB1/alkM* transcripts, which encode PE-degrading enzymes, and transporters such as *fadL, livG, livF, livH,* and *livM* were upregulated, along with transcripts associated with the fatty acid β-oxidation pathway.

**Conclusions:**

In this study, we address the underlying patterns of gene expression during biofilm development in a PE-associated plastisphere in soil and address the pressing question of whether natural microbial communities have the potential to biodegrade petrochemical-based plastic in the soil environment.

**Supplementary Information:**

The online version contains supplementary material available at 10.1186/s40793-024-00621-1.

## Background

Plastic has become prevalent in the environment because of various human activities. It is known to accumulate as a pollutant in terrestrial ecosystems through a variety of sources, such as improper disposal, blow-offs from landfills, and large remains of mulching foils and sewage sludge used in agricultural applications [1, 2]. Its existence in the soil is presently approximated to be 4–23 times greater than that in the ocean. Since soil microorganisms are typically the initial responders to human-caused disturbances, their interactions with plastic debris are vital for the resilience of soil ecosystems [3]. Recent studies have shown that plastic debris can shape the associated soil microbial community similarly to the microbial communities found on plastic debris in aquatic ecosystems, which characterize the terrestrial plastisphere as a distinct microbial habitat [4–10].

Once discarded to the ground and integrated into the soil matrix, plastic debris can jeopardize several crucial healthy soil characteristics, such as soil aggregation [11], hydraulic conductivity and water-holding capacity [12]. It also has adverse dose-dependent impacts on agricultural crop growth [13, 14]. The majority of plastic waste is produced in the packaging sector, in which polyethylene constitutes the largest portion of plastic primary production [15]. In 2015, 97 million tons of polyethylene (PE) were disposed of either into landfills or incinerators or eventually entered natural environments [16]. PE is also commonly utilized as mulching film or foil in agricultural fields, where fragmented PE residues have accumulated in soil over the years, posing a tremendous threat to global soil health [17, 18].

PE is a petrochemical-based polymer consisting of long chains (~ 4000–40 000 carbon atoms) of -CH_2_--CH_2_- functionalities derived from ethylene monomers whose carbon backbone is either branched, as in low-density polyethylene (LDPE), or linear, as in high-density polyethylene (HDPE). Owing to their high molecular weight and hydrophobicity, successful microbial degradation requires organisms to possess cellular properties that allow interactions with recalcitrant PE surfaces [19]. Although a surprisingly large number of bacterial and fungal genera are associated with PE degradation (*Acinetobacter*,* Arthrobacter*,* Aspergillus*,* Bacillus*,* Brevibacillus*,* Klebsiella*,* Nocardia*,* Pseudomonas*,* Rhodococcus*,* Staphylococcus*, and *Streptomyces*) [19–23], only a few studies have proposed biochemical mechanisms or enzymes expressed by PE-associated microorganisms [24–26]. Studies that screen microbial metagenomes for potential plastic-degrading enzymes from uncultivated microorganisms have demonstrated the great potential of meta-omics techniques to elucidate the underlying mechanisms of PE degradation [27, 28].

Crucial for microbial colonization of plastics and potential degradation, is an initial abiotic deterioration driven by UV radiation, heat, and mechanical forces which lead to macromolecular changes in the polymer structure and an increase in the number of hydrophilic groups on the surface [29]. This creates favorable conditions for plastic-associated biofilm formation. Owing to the complexity of the enzymatic processes involved in the complete biodegradation of long-chained polymers such as PE, it must be assumed that different microorganisms are responsible for the various steps of fragmentation and deterioration of the plastic material up to the assimilation and mineralization of plastic-derived carbon [30–32]. The development of mixed microbial biofilms was observed to play a significant role in breaking down plastics in soil environments, encouraging direct contact and potential degradation processes at the biofilm‒plastic interface [33].

Like the formation of biofilms, the colonization of plastic progresses through different successional stages: (i) In the initial attachment phase, pioneering microorganisms that possess physiological features to adhere to the plastic surface (e.g., high cell hydrophobicity) rapidly colonize the plastic surface [34]. During this stage, the physicochemical surface properties of the polymer, such as the polymer type, hydrophobicity, roughness, and degree of surface weathering, play a major role [35]. Research indicates that following this initial attachment phase, a selection phase (ii) ensues in which plastic-degrading taxa (e.g., *Rhodococcus*,* Micrococcus*,* Pseudomonas*, and *Brevibacillus spp*.) will be enriched and compete for the released PE oligomers and/or leaching additives as a carbon source from the plastic itself [36–38]. Extracellular enzymes such as hydroxylases, oxygenases, laccases, and other candidate enzymes cleave the polymeric backbone into smaller oligomers, such as aliphatic carboxylic acids, aldehydes, and alkanes, which can later be assimilated and metabolized by bacteria or fungi [39, 40]. As the biofilm matures further, more cells grow as a secondary biofilm layer, forming irreversibly attached cell aggregates with the excretion of extracellular polymeric substances (EPSs), which stabilize and protect multicellular communities. Throughout this mature successional phase (iii), the plastic-derived carbon sources are exhausted (e.g., low-molecular-weight polymer fragments, carboxylic acids, etc.), and the polymer properties no longer play a major role in shaping the microbial community. Phototrophy, grazing, and cross-feeding have become the major fuels for microbial growth, which outcompete pioneering specialized plastic-associated taxa [41–44].

In our study, we aimed to track the changes in both microbial taxonomy and the expression of PE-associated genes within a biofilm community formed on soil-buried HDPE foil (hereafter PE foil) during all successional phases. To do so, we performed a soil burial experiment with pieces of UV-weathered PE foil in soil microcosms filled with two types of soil: (A) soil from an abandoned landfill high in plastic content (plastic soil) and (B) soil that has been exposed to less plastic pollution (forest soil). Total RNA was extracted directly from the colonized surfaces at four time points (T_1_: 2 days, T_2_: 7 days, T_3_: 14 days, and T_4_: 53 days) and used for a metatranscriptome analysis of both the biofilm taxonomy and a functional analysis of potential genes involved in PE degradation. As early biofilm succession selects microorganisms capable of utilizing the polymer as a substrate for growth, we expected enrichment of microbial taxa and genes associated with PE degradation to occur within the early sampling points. Previous studies have indicated that once in contact with the soil microbiome, plastic debris enriches a specialized microbial community, which is distinct in its taxonomic composition and adaptation to plastic as a substrate [7, 9, 10, 45].

We therefore hypothesized that microbial communities with greater exposure to plastic debris in their soil matrix would likewise colonize and possibly utilize a newly introduced PE foil more efficiently. We predicted that the landfill microbial community would be more effective than the forest soil community in terms of colonizing the PE surface and expressing a greater variety of genes and taxa related to PE degradation. Furthermore, by imaging biofilm succession by scanning electron microscopy (SEM), we compared surface interactions and biofilm coverage on both glass and PE during the course of biofilm succession. In this study, we aimed to elucidate the underlying patterns of gene expression that take place during the biofilm development of a PE-associated plastisphere in soil. Furthermore, in light of ever-increasing plastic production worldwide, our research addresses the pressing question of whether natural microbial communities have the potential to biodegrade petrochemical-based plastics in the soil environment.

## Materials and methods

### Material and UV-weathering of HDPE foil

The HDPE polyethylene foil (hereafter PE) used for the incubation experiments was kindly provided by Bundesanstalt für Materialforschung und -prüfung (BAM), Berlin. To mimic outdoor weathering conditions and increase the susceptibility of the foil to microbial attachment, the PE foil was UV-weathered for 100 h at 75 °C in a UV weathering chamber (Global-UV Test 200, Weiss Umwelttechnik, Vienna, Austria) with UV-fluorescence lamps according to DIN EN ISO 4892-3, type 1 A (UVA-340). The HDPE samples had a total surface energy of 14 MJ m^− 2^ (40 W m^− 2^ × 100 h). The glass slides used as nonplastic controls were standard square cover glass slips (BRAND^®^, Germany) with dimensions of 1.8 cm × 1.8 cm.

### Study design and incubation

To incubate PE foil and glass slides in controlled soil microcosms, we sampled soil from a forest and an abandoned landfill with highly plastic polluted soil (hereafter plastic soil) from the following two locations:

The forest soil was sampled from a fenced forest area in Potsdam-Süd, Germany (52°22’48.8"N, 13°03’34.4"E), from which approximately 100 g of the topsoil layer (< 10 cm) was sampled at four random sampling locations within a 10 m radius. The plastic soil characterized by a high plastic content was sampled at an abandoned landfill in Niemegk, a town in the Potsdam-Mittelmark district of Brandenburg, northeastern Germany (52°02′58.8″ N 12°39′34.8″ E). We collected samples from four different locations on the landfill within the topsoil layers (< 10 cm) and identified soil samples with high plastic contents both by visual inspection and mass thermogravimetry-mass spectrometry (TGA-MS) from the sampled soils (Samples NGK in Figure S1 of [10]). Both soil types were sampled in the summer months (August 2018, September 2021), stored in sterile glass bottles and frozen at -20 °C until further handling. The soils were thawed over a week at increasing temperatures from 8 °C to 16 °C in the dark to acclimate and re-establish microbial growth. The four soil subsamples from each of the two locations were pooled and mixed uniformly into one sample, from which 30 g of material was placed in a sterile glass bottle (volume 100 ml) and wetted with demineralized water at 60% water holding capacity (WHC). The bottles were lifted horizontally to increase the soil surface area to its maximum value. In each bottle, three pieces of PE foil and three glass slides were carefully placed and buried with soil material. In half of the bottles, one extra PE foil/glass slide was placed for SEM analysis. Before soil burial, we surface disinfected both the PE foil and the glass slides with 70% ethanol for 15 min. The PE foil was cut into pieces of approximately 1.5 × 1.5 cm. Bottles were incubated over a period of T_1_: 2 days, T_2_: 7 days, T_3_: 14 days 4 and T_4_: 53 days at 28 °C with LED light (SanLight model S2W), which mimicked a 6-hour light/dark cycle.

### SEM sampling and fixation

For SEM analysis of biofilms, one glass slide and one piece of PE foil per time point were gently rinsed with demineralized water before being fixed in Methacarn (60% methanol, 30% chloroform, and 10% acetic acid) solution for at least 24 h. After fixation, the samples were dehydrated in a graded series of 30% (5 min), 50% (10 min), 70% (15 min), or 99% (20 min) ethanol according to the adjusted protocol of Dassanayake et al., 2020. The samples were coated with 12 nm of carbon via a Leica EM ACE600 coater. SEM imaging was performed on an FEI Quanta 3D SEM at an accelerating voltage of 10 kV, beam current of 23–93 pA, and working distance of 6–10 mm via a secondary electron detector. To compare surface coverage, four images per time point and type of soil on both PE and glass were captured and analyzed. The surface coverage, cell morphology, and general organic features of the different soils and time points were documented and compared.

### RNA and DNA extraction

At each time point, two bottles per soil type (biological duplicates) were sampled destructively and handled under sterile and nuclease-free conditions. Out of each bottle, PE foils (*n* = 3) and glass slides (*n* = 3) were carefully recovered with sterilized metal tweezers and rinsed with nuclease-free water (sterile DEPC-treated water, Carl Roth GmbH) to remove external soil particles. PE foil and glass slides were weighed and placed directly into ZYMO Research Bead Lysis tubes for immediate RNA extraction via the ZYMO Research *Quick-*RNA ^TM^ Soil Microprep Kit. The extraction samples were lysed and homogenized with a FastPrep-24™ beating grinder at two cycles of 25 Sect. (6 m s^− 2^) and placed on ice immediately after. The extracted RNA was eluted in 15 µL of nuclease-free water and stored at -80 °C before the total RNA yield was quantified.

For metagenome sequencing of the bulk microbial communities present in the two different soils, we collected DNA samples from each bulk material (plastic soil or forest soil) before incubation at timepoint T_0_ and after 53 days of incubation at the final timepoint T_4_. DNA extraction was performed via a Roboklon EurX Soil DNA Purification Kit with 250–300 mg of bulk soil material per sample, and the samples were further handled according to the protocol below.

### Quality control and metatranscriptome/metagenomic sequencing

To remove any residual DNA from the RNA extracts, the eluted RNA samples were treated with DNAse via a TURBO DNA-free kit (Thermo Fisher Scientific, Germany) before further quantification and library preparation. All RNA samples were then analyzed via a Bioanalyzer RNA 6000 Nano Assay to determine yield and integrity. RNA extracts from each triplicate from a single bottle were pooled and concentrated to a final concentration of 150 ng of RNA in 15 µL via vacuum centrifugation (Concentrator plus, Eppendorf, Germany) for metatranscriptome sequencing. In this way, we obtained biological duplicates per time point and soil type for further sequencing. Library preparation and RNA/DNA sequencing were performed by Eurofins Genomic Germany GmbH (Anzinger Str. 7a, 85560 Ebersberg, Germany) on an Illumina NovaSeq 6000 platform aiming for 30 million paired-end reads of length 150 nt. The final sequencing depth differed between samples since the obtained RNA concentrations were variable and generally low due to the limitation of attached biomass, especially during early biofilm succession. To compensate for the overall low biomass of the attached biofilms, we used triplicate samples from each experimental unit and pooled their RNA yield as described above. All the raw reads were subsequently processed via the ATLAS metagenome pipeline (QC module) to obtain dereplicated, quality-controlled, and trimmed reads [46].

### De Novo assembly of metagenomic reads and functional annotation

The ATLAS metagenome pipeline (module assembly) was used to generate contigs from the DNA reads [46]. From these contigs, genes were predicted via prodigal v2.6.3 [47] and annotated via eggNOG emapper v2.0.1 [48] with a database from October 2020 [49]. This resulted in contigs and annotated genes for each of the 4 DNA samples (forest/soil for 2d and 53d).

### Taxonomic assignment and KEGG annotation of RNA reads

The RNA reads were mapped to the DNA contigs (53 days of the corresponding bulk soil), and the reads were counted per gene. The obtained counts were normalized as follows: First, gene counts were corrected for sequencing depth with Bowtie2 v2.4.2 [50] by normalization to the total mapped sequencing reads of each sample (per million mapped reads). Second, to correct for different gene lengths, counts were normalized to kilobases of gene length (e.g., for a gene with a length of 2000 nt, the count was divided by 2). These normalized gene counts are comparable between different genes within and between samples. The functional annotation with EGGNog (see above) also annotates KEGG IDs. To obtain summarized KEGG counts, the normalized counts were simply summed by the KEGG ID. To obtain a detailed taxonomic assignment of the active microbial species, quality-controlled 16 S/18S rRNA/DNA reads were mapped to the SILVA SSU database v138 [51] to calculate the abundance of microbial ASVs in the plastisphere community. Mapping was performed via Bowtie2 v2.4.2 [50]. Taxon abundance was finally obtained by summing gene counts by taxonomic level of interest. For the functional profiling of active transcripts within the plastisphere community, we used our KEGG-annotated metatranscriptomic data and compared them to a set of 35 known enzymes involved in PE and/or general polymer colonization, five genes related to nitrogen fixation (*nif* genes) and 133 biofilm-related enzymes involved in lipopolysaccharide biosynthesis and extracellular matrix synthesis (Supplementary Table 2) [20, 25, 52].

### Data analysis

Data analysis and plotting were performed via RStudio version 2022.07.0 + 548 “Spotted Wakerobin” release for Ubuntu Bionic. The R packages used for sequence data analysis included vegan, phyloseq, and gene filter. We performed SEM image analysis and calculation of the surface coverage with Fiji ImageJ version 1.53q using a Kuwabara filter (radius 3) and the Triangle algorithm for threshold analysis of biofilm coverage. The graphics were adjusted via Inkscape version 0.92.5.

## Results

### RNA sequencing and taxonomic annotation

**Fig. 1:**
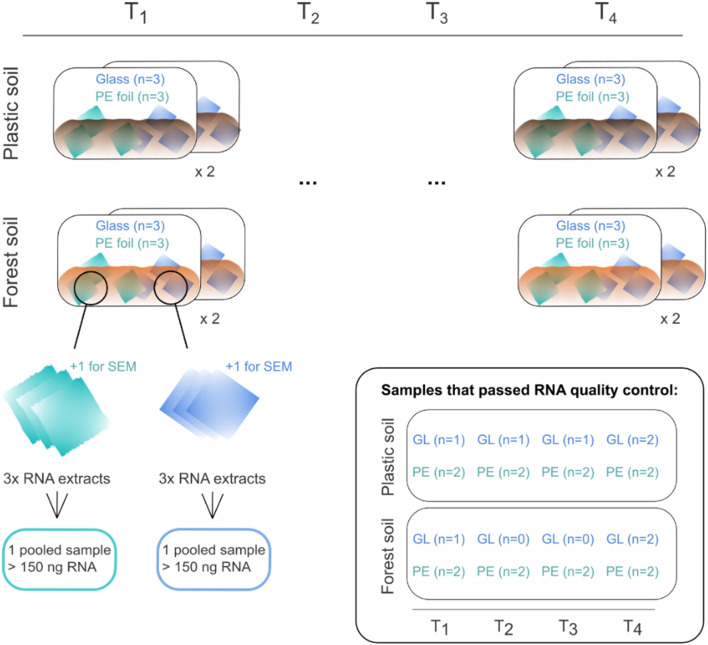
Experimental setup of soil microcosms and RNA extraction scheme for both soil types

A total of 263 059 818 raw reads were obtained from 24 pooled RNA samples (plastic soil: 8 PE samples and 5 glass samples; forest soil: 8 PE samples and three glass samples), which ultimately passed quality control (average of 28.32 ng RNA per sample), with an average of 10 960 826 reads per sample (Fig. 1).

After merging and removing the chloroplast and mitochondrial reads, we obtained a total of 15 029 taxonomic units from our RNA-Seq library, from which 1 059 of the taxonomic units were filtered out due to low relative read number abundances (read number mean abundance < 10^−5^). The microbial diversity and community structure were thus analyzed on the basis of a total of 9 594 prokaryotic and 4 376 eukaryotic taxonomic units (Supplementary Table 1).

Soil- and time-dependent biofilm succession on PE and glass.

**Fig. 2:**
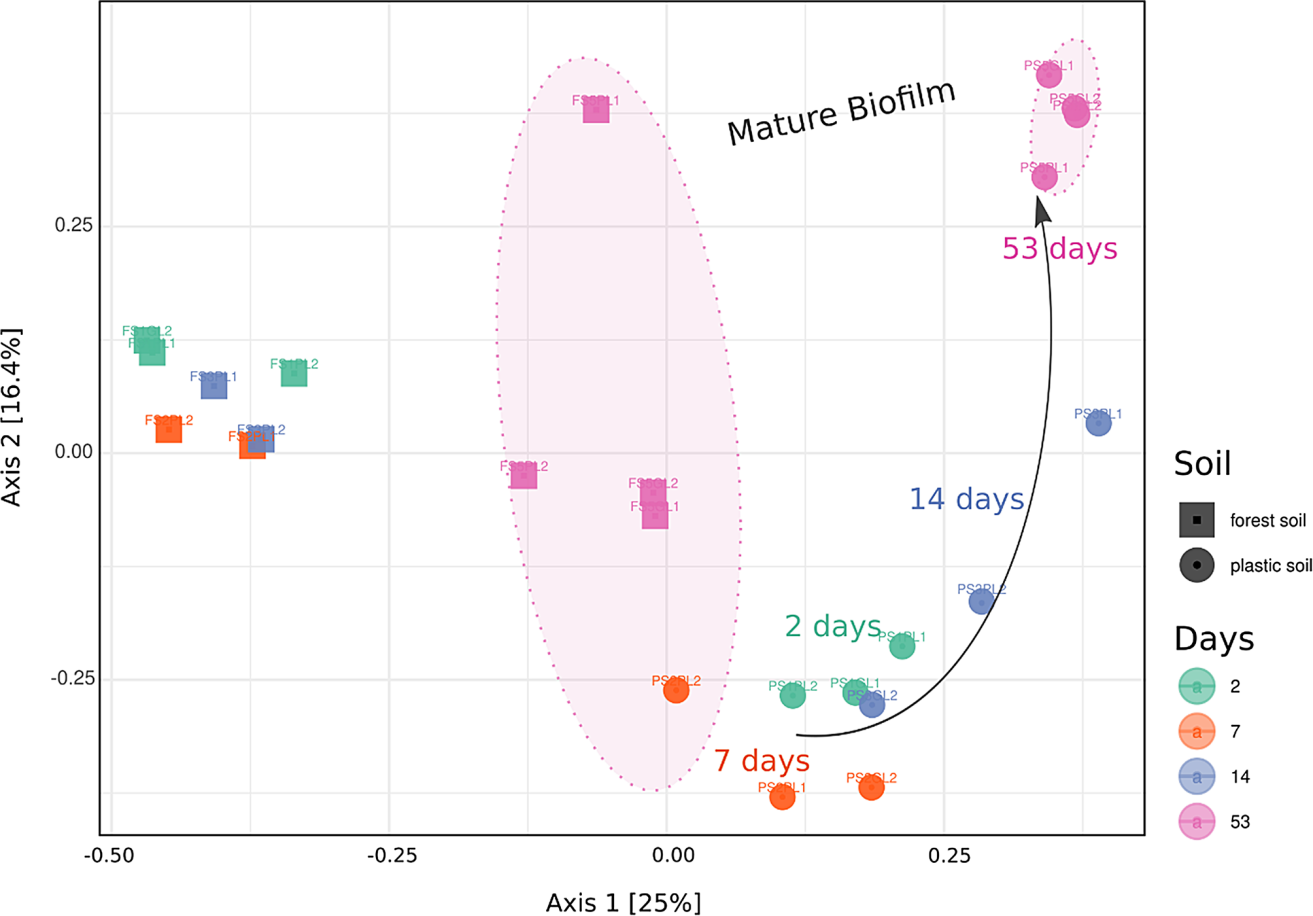
Principal coordinate analysis (PCoA) of distances between the PE/glass biofilm community structures in two soil types. The data were clustered on the basis of the type of soil (R²= 0.213, *p* = 0.001) and the successional stage of the biofilm (R²=0.246, *p* = 0.002). Ordinations are based on Bray–Curtis measures of dissimilarity, and ellipses represent 95% confidence intervals. The biofilm community structures from the plastic soil clearly separated with increasing incubation time, whereas those from the forest soil remained more similar over time. The mature biofilm communities (day 53) clustered separately in both soil types

To compare different successional stages of biofilm growth on PE vs. glass, we used the taxonomic composition assigned from our RNA transcripts and calculated their relative abundance within the active portion of the sequenced community to generate a PCoA plot based on Bray‒Curtis measures of dissimilarity (Fig. 2).

According to the PERMANOVA results, the type of soil explained 21% of the observed differences between the biofilm communities (Table 1), whereas the type of substrate (PE vs. glass) did not contribute to a significant shift in the biofilm community structure (R^2^ = 0.05, *p* = 0.266).

**Table 1:** PERMANOVA testing of factors explaining differences in the biofilm community

	df	Sums of square	Pseudo F	*R*²	*P* value	Significance
**PE vs. glass**	1	0.4566	1.2581	0.05	0.266	-
**Forest soil vs. Plastic soil**	1	1.8062	5.9899	0.21	0.001	***
**Days**	3	2.0328	2.1151	0.24	0.002	**
**Early biofilm (Day 2 vs. rest)**	1	0.5118	1.4202	0.06	0.152	-
**Early mid biofilm (Day 7 vs. rest)**	1	0.7724	2.2162	0.09	0.015	*
**Late mid biofilm (Day 14 vs. rest)**	1	0.3082	0.8338	0.04	0.584	-
**Late biofilm (Day 53 vs. rest)**	1	1.1902	3.6116	0.14	0.001	***

If a p value is less than 0.05, it is flagged with one star (*). If a p value is less than 0.01, it is flagged with 2 stars (**). If a p value is less than or equal to 0.001, it is flagged with three stars (***)

We observed a clear separation of the sequenced biofilm communities incubated in plastic soil *versus* those incubated in forest soil, which deviated along the primary principal axis (Fig. 2). PERMANOVA revealed that the type of soil explained 21% of the observed differences between the biofilm communities. Within the two soil types, the most apparent separation of the microbial communities was explained by the different successional stages of the biofilm according to the number of days of incubation (Fig. 2). In both the plastic soil and the forest soil, the microbial communities changed during biofilm progression from early biofilm (days 2–7) to a mid- (Day 14) and late biofilm succession (day 53). The most advanced point of our biofilm incubation experiment (Day 53) also presented the most distinct microbial beta diversity, which differed significantly from the other sequenced time points in both forest and plastic soils (PERMANOVA: R² = 0.146, *p* = 0.0012).

Most striking was the community succession of the incubated biofilm in plastic soil. All four time points formed individual clusters along the two axes of the PCoA (Fig. 2), and the development of the biofilm communities was observed on the basis of their taxonomic shifts from early to mature colonization. In contrast, the community structure of the biofilms in the forest soil clustered closely together during the early- and mid-biofilm successional stages and showed a significant shift in community structure only on day 53 (Fig. 2).

**Fig. 3:**
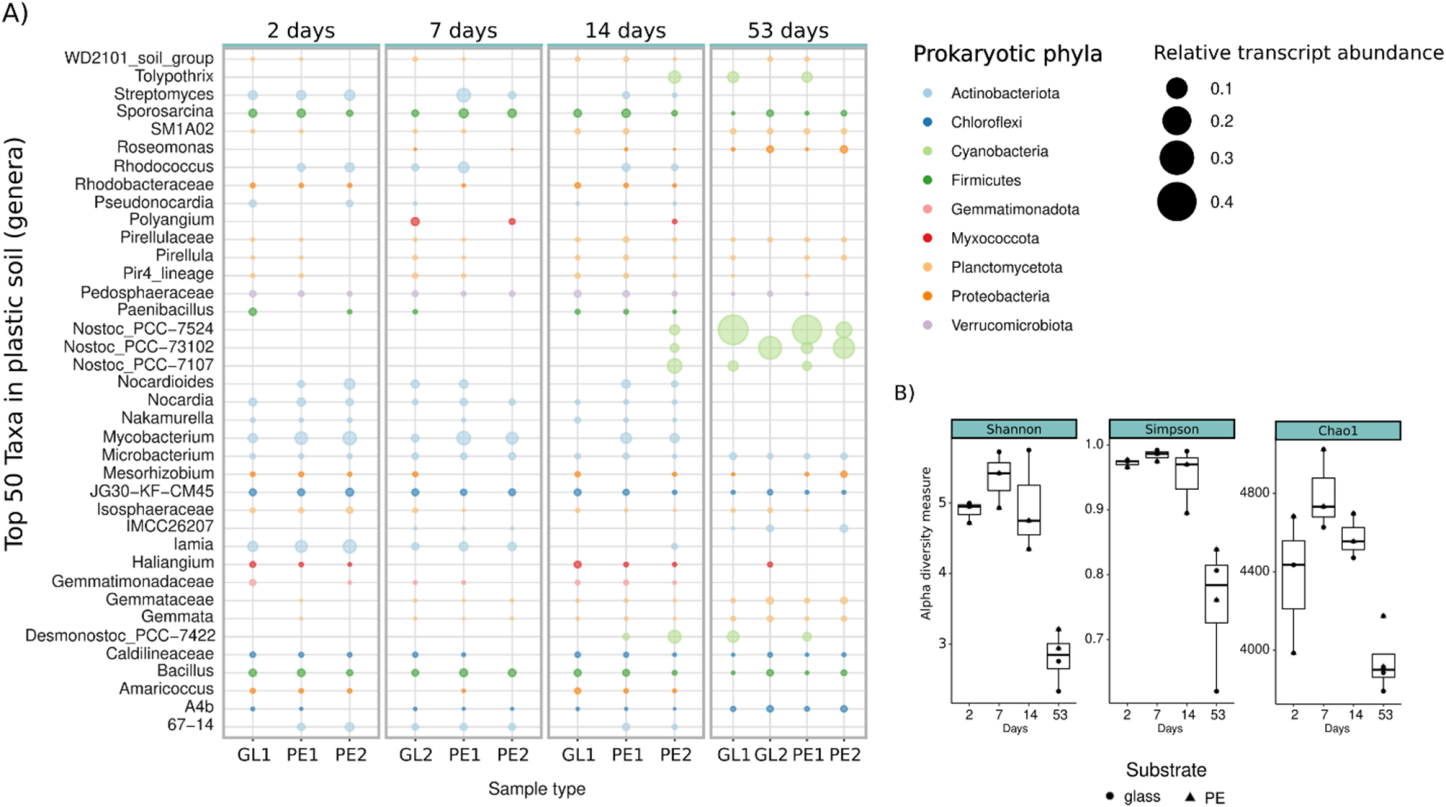
Taxonomy and alpha diversity of the bacterial biofilm community in plastic soil. The relative abundances of the 50 most abundant bacterial ASVs are shown at the genus level **(A)**. The rows show their corresponding genera, and the colors represent their phyla. The size of the bubbles depicts the relative SSU rRNA abundance in the metatranscriptome. The X-axis labels show PE for polyethylene, GL for glass and numbers 1 and 2 correspond to the biological replicates. The plot shows a clear change in the relative abundance of SSU rRNA over time with increasing numbers of phototrophic organisms. **(B)** Decrease in bacterial alpha diversity, as calculated with the Chao1 index of species richness, the Shannon index of diversity (H’) and the Simpson index of dominance (D’). Means not sharing any letter are significantly different according to the Tukey HSD test, with *p* < 0.05. Boxes span the interquartile range (IQR), with the middle line being the median of the corresponding indices

**Fig. 4:**
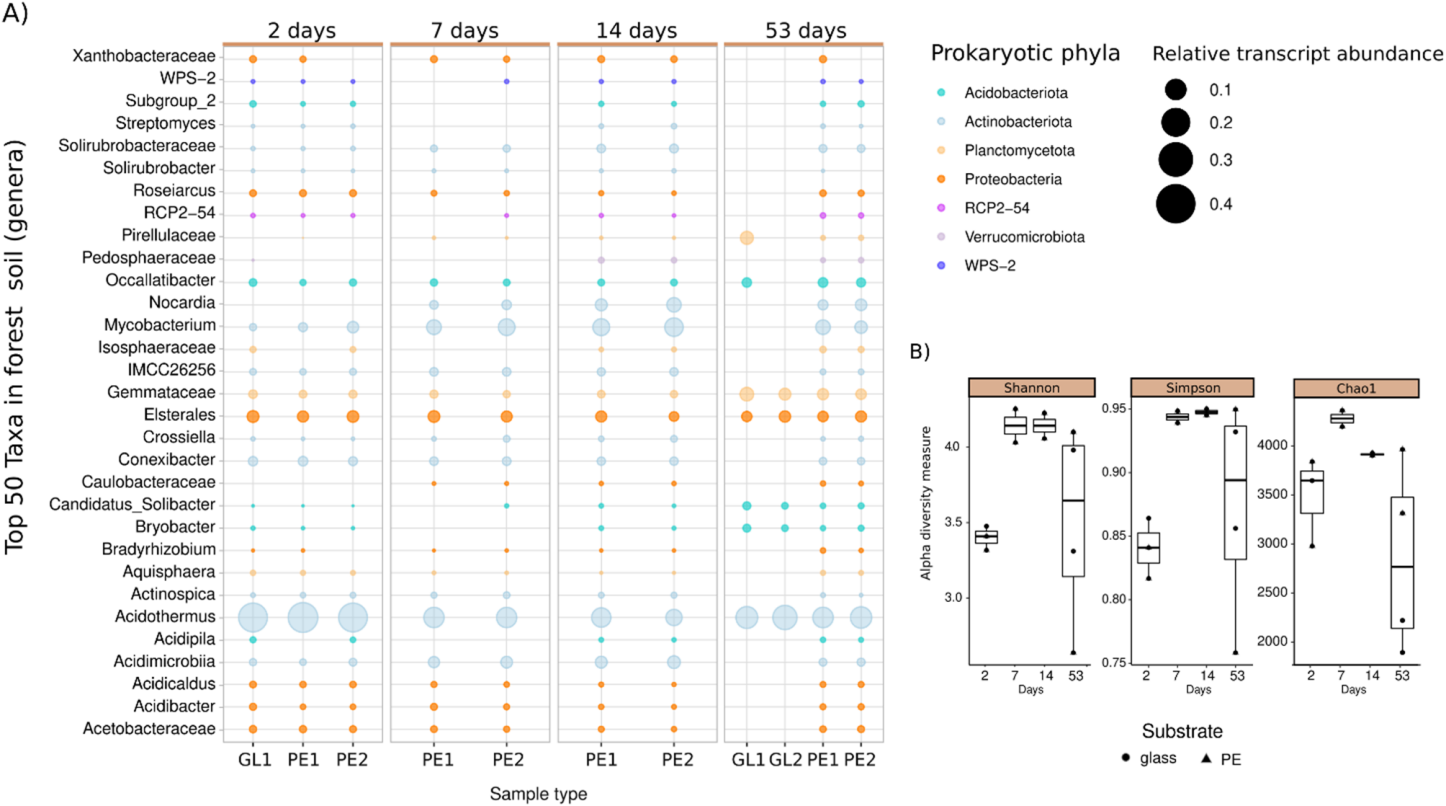
Taxonomy and alpha diversity of the bacterial biofilm community in forest soil. The relative abundances of the 50 most abundant bacterial ASVs are shown at the genus level **(A)**. The rows show their corresponding genera, and the colors represent their phyla. The size of the bubbles depicts the relative SSU rRNA abundance in the metatranscriptome. The X-axis labels show PE for polyethylene, GL for glass and numbers 1 and 2 correspond to the biological replicates. The changes in bacterial composition and activity are less pronounced than those in plastic soil (Fig. 2). In addition, no Cyanobacteria were observed. **(B)** did not significantly decrease bacterial alpha diversity, as calculated with the Chao1 index of species richness, the Shannon index of diversity (H’) and the Simpson index of dominance (D’). Means not sharing any letter are significantly different according to the Tukey HSD test, with *p* < 0.05. Boxes span the interquartile range (IQR), with the middle line being the median of the corresponding indices

**Fig. 5:**
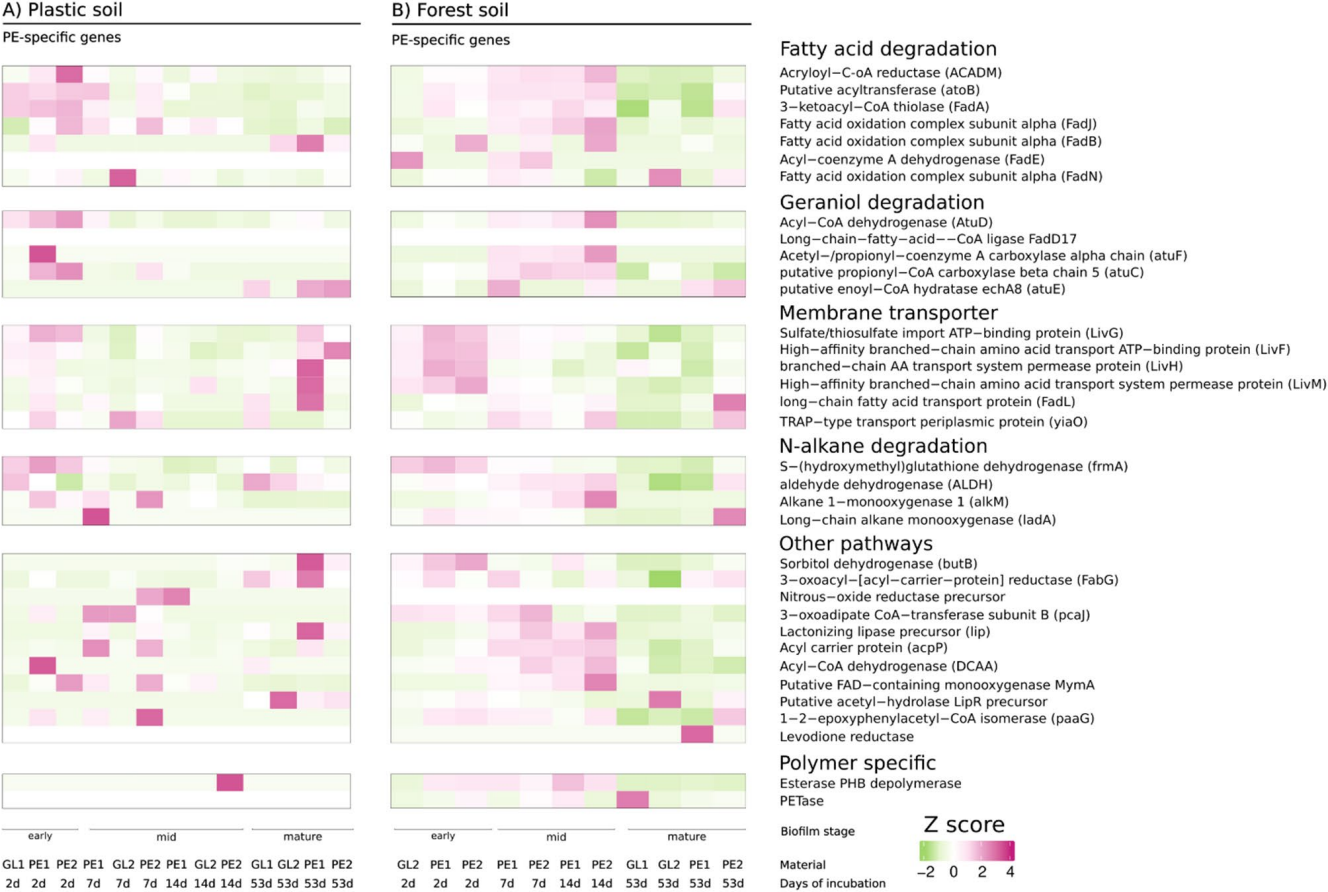
Gene expression levels of PE-specific genes present in the biofilm communities of forest soil **(A)** and plastic soil **(B)** over time. The gene expression counts of PE-specific genes were normalized via Z score transformation and grouped according to metabolic function. Sample replicates per day are depicted as GL (glass) and PE (PE foil)

**Fig. 6:**
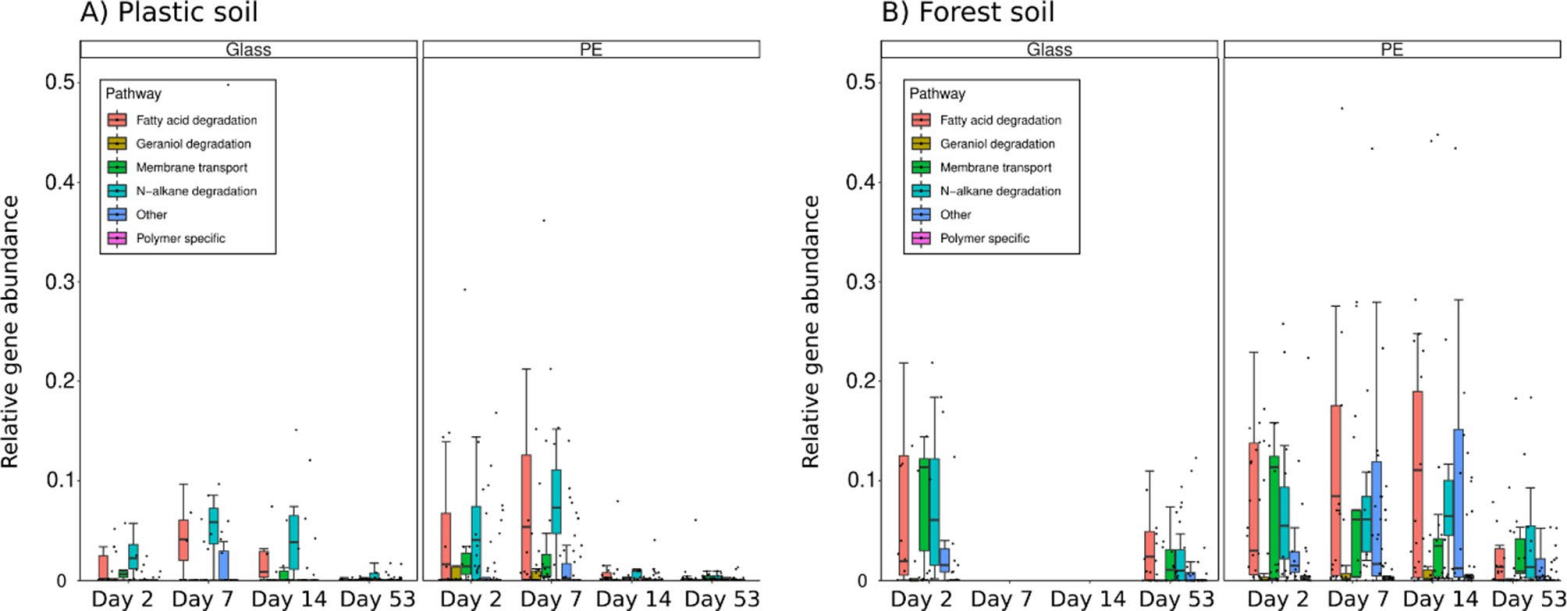
Relative gene expression of PE-specific genes over the course of 53 days on PE vs. glass surfaces in **(A)** plastic soil and **(B)** forest soil. Observations are depicted as single dots, and boxplots show medians with interquartile ranges (IQRs) grouped by metabolic function. Missing observations were due to low RNA yield during the extraction phase. The forest soil samples presented overall greater gene expression during the incubation phase, whereas the PE-specific gene expression in the plastic soil decreased during day 14 of biofilm growth

To test the activity and function of the biofilm microbial taxa, we compared their expression levels of 16 S/18S rRNA gene transcripts for taxonomic assignment (Figs. 2, 3 and 4) at the given points in time as well as the expression levels of specific genes relevant for the PE-plastisphere (Supplementary Table 2) for functional profiling (Figs. 5 and 6). By using this metatranscriptomic approach, we were able to follow only the active portion of the biofilm community over 53 days in the two different soils.

### Plastic soil

Compared with those in the initial successional stage, the PE-associated biofilm communities in the plastic soil developed over the course of time into a less diverse community (Fig. 3). After an initial increase in diversity on day 7 (median _Chao1_ = 4732.7, median _H’_ = 5.42, median _D’_ = 0.99), the bacterial diversity decreased significantly within the biofilm of the late successional stage on day 53 (median _Chao1_ = 3899.8, median _H’_ = 2.85, median _D’_ = 0.78). We found no significant differences in alpha diversity between the biofilm communities on PE and those on glass at any successional stage. However, concerning the taxonomic makeup of the attached microbial communities, we did observe slight differences between the microbial community attached to PE and the biofilm community attached to glass (Fig. 3). A preference of known plastic-associated taxa (e.g., *Rhodococcus*, *Nocardioides* and *Streptomyces*) for PE surfaces was observed in the early- to mid-biofilm stages compared with glass; however, none of them were exclusively active on PE surfaces (Fig. 3).

In the early- to mid-biofilm stages (between 2 and 14 days), transcripts of the Actinobacteria and Firmicutes were the most abundant prokaryotic phyla of the biofilm communities in the plastic soil (Fig. 3). Transcripts of the genera *Lamia* (4.9% mean relative abundance), *Mycobacterium* (3.3%), *Nocardioides* (2.2%), *Rhodococcus* (2.4%) and *Streptomyces* (2.0%) were dominant in the early stages of biofilm succession, followed by the members of the *Firmicutes* phylum *Sporosarcina* (1.4%) and *Bacillus* (1.1%). All of them decreased in their SSU rRNA abundance during biofilm succession (Fig. 3) and disappeared in the late biofilm stage (e.g., *Proteobacteria*) or were otherwise present in abundances below 0.1% (*Firmicutes*). Other SSU rRNA transcripts present in early biofilm succession included *Chloroflexia JG30-KF-CM45* (2.0%) of the Chloroflexi phylum and *Pedosphaeraceae* of the Verrucomicrobiota phylum (1.2%). Proteobacteria were represented by transcripts of the genera *Amaricoccus* (0.7%), *Mesorhizobium* (0.8%) and *Rhodobacteraceae* (0.6%).

The active eukaryotic fraction of the biofilm community consisted of the Ascomycota *Aspergillus* (0.9%), single-celled protists such as Cercozoa (1.0%) and Cercomonas (0.3%) (Supplementary Figure S1 A). Other abundant eukaryotic members of the community included the saprotrophic fungus *Mortierella* (1.8%), the protozoa (*Ciliophora*) and the order *Agaricales* (gilled mushroom). Trancriptomic reads originating from *Embryophyta* (plants) and *Acanthamoeba* (amoeba) were likewise present across all the communities (Supplementary Figure S1 A).

Furthermore, the most apparent shift in the activity of the microbial community was detected between days 14 and 53 of biofilm succession. The transition from a mid-succession stage to a late biofilm stage was accompanied by a strong increase in Cyanobacterial transcripts (up to 49% of all sequenced reads per sample) and a decrease in other early-to-mid-biofilm members (Fig. 3). The autotrophic genera of Nostocaceae, e.g., *Nostoc_PCC* and *Tolypthrix*, were highly abundant. Other eukaryotic late colonizers were the green algae *Chlorophycae* (7%) and *Trebouxiophycae* (2.5%) of the Chlorophyta phylum (Supplementary Figure S1). The Firmicutes in the late biofilm succession were *Bacillus* (1.0%) and *Sporosarcina* (0.9%), whereas the Chloroflexi phylum was represented by the genera A4b of Anaerolineae (1.3%), *JG30-KF-CM45* (0.6%) and Caldilineaceae (0.5%). Additionally, phylum members of Planctomycetota were a dominant part of the late biofilm community, with Gemmataceae (1.24%), SM1A02 (1.2%) and Pirellulaceae (0.67%) being dominant. Among the Actinobacteriota, only Microbacterium (1%) and Microtrichaceae (1.5%) were present at the late successional stage. More highly abundant eukaryotic transcripts were again derived from *Embryophyt*a (17%) and reads of the grazing protists *Acanthamoeba (1%)* an*d Vermamoeba (0.6%).*

### Forest soil

Similar to the results for the biofilm communities in the plastic soil, we first observed a slight increase in diversity peaking on day 7 of incubation (median _Chao1_ = 4279_,_ median _H’_ = 4.14, median _D’_ = 0.94) and a gradual decrease in diversity toward the late successional stage after 53 days (Fig. 4). The differences in forest soil were, however, smaller than those in plastic soil, and none of them were statistically significant (Fig. 4). The sequenced biofilm community in forest soil remained more stable overall during biofilm succession, and the taxonomic differences were also less distinct between the different biofilm stages (Fig. 4).

The early biofilm community was again dominated by Actinobacteriota such as *Acidothermus* (32.9%), *Mycobacterium* (6.9%), *Conexibacter* (3.5%) and *Acidimicrobiia* (3.6%), which were the top initial colonizers in the biofilm community on both substrates (Fig. 4). However, the early biofilms derived from forest soil contained more members of Proteobacteria, such as *Elsterales* (5.9%), *Acetobacteraceae* (1.9%) and *Roseiarcus* (1.6%), as well as members of the phylum Planctomycetota, e.g., the cellulose degrader *Gemmataceae* (2.6%).

Compared with those in plastic soil, there were fewer taxonomic changes during biofilm succession until day 53 of incubation in forest soil. During the late succession stage of the biofilm, there was a tremendous decrease in taxonomic richness on glass compared with that on PE (Fig. 4 and Figure Supplementary Figure S1 B). Both the bacterial and eukaryotic fractions of the biofilm on glass consisted of a few highly abundant taxa. The Actinobacteriota *Acidothermus* was highly abundant, accounting for up to 43.4% of all reads in a given sample. Equally abundant were the genera *Gemmataceae* and *Pirellulaceae* of the Planctomycetota phylum. A reduced taxonomic diversity on glass-associated biofilms was mostly visible in the top 50 prokaryotic taxa (Fig. 4), as only seven of the top genera were present in the mature biofilm (day 53) on glass, whereas 31 unique genera of higher abundance were present within the PE-associated biofilm on day 53.

With respect to the eukaryotic fraction of the biofilm community, fungi were more abundant in forest soil than in plastic soil. The genera *Aspergillus* (3.4%) and *Penicillium* (5.3%) of the phylum Ascomycota were abundant in the early- to mid-successional stages of the biofilm community but decreased in abundance within the mature biofilm community (Supplementary Figure S1 B). In contrast to the eukaryotic community in plastic soil, we detected no members of the *Chlorophyta* phylum (green algae) in the early- to mid-biofilm stage but strongly dominated the late biofilm stages by these genera (Supplementary Figure S1 B). The changes in bacterial composition and activity were less pronounced than those in plastic soil. In addition, no Cyanobacteria were detected in the forest soil biofilm community.

### PE-specific gene expression marks successional biofilm stages on PE and glass

**Fig. 7:**
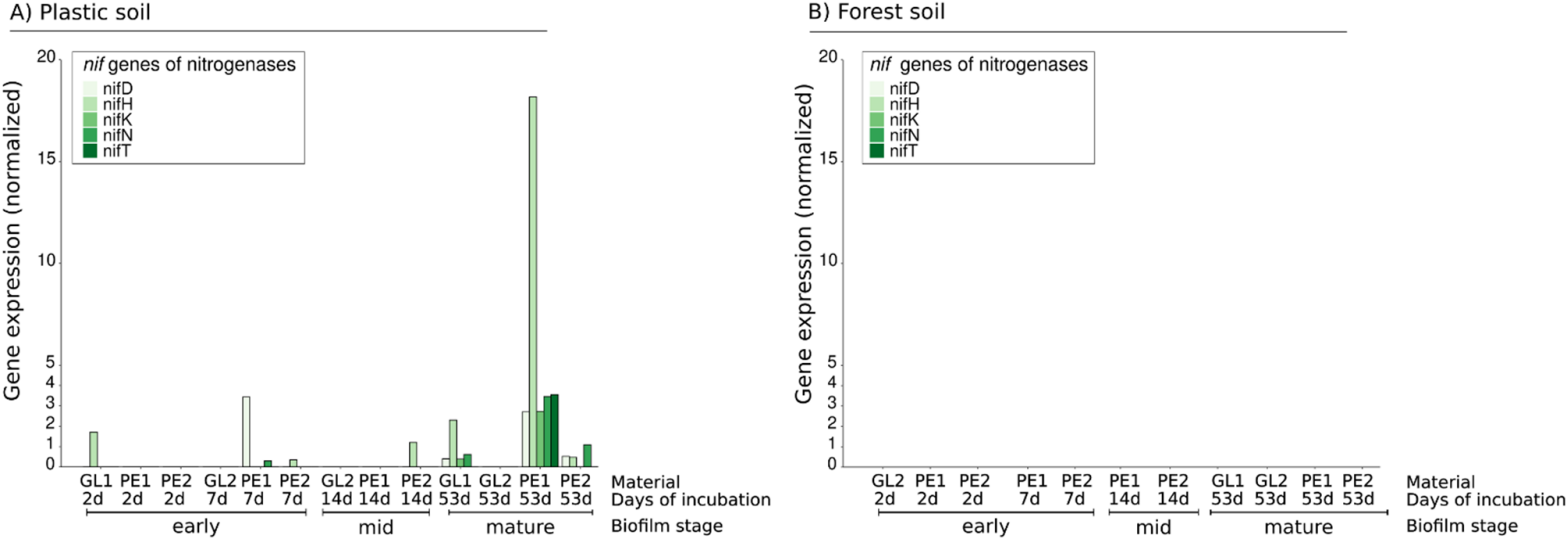
Relative gene expression of the *nif* gene family responsible for nitrogen fixation over the course of 53 days in **(A)** plastic soil and **(B)** forest soil. The bars show the relative gene expression of the KEGG entries K02586 (*nifD*), K02586 (*nifH*), K02591 (*nifK*), K02592 (*nifN*) and K02593 (*nifT*) in each of the biofilm samples. None of the genes were expressed in b) forest soil

To identify specific pathways involved in the succession of PE-associated biofilms, we used our KEGG-annotated metatranscriptomic data and compared them to a set of 35 known genes involved in PE and/or general polymer colonization (Figs. 5 and 6), five *nif* genes related to nitrogen fixation (Figs. 7) and 133 biofilm-related genes, which encode enzymes involved in lipopolysaccharide biosynthesis and extracellular matrix synthesis (Supplementary Figure S2). The gene expression patterns of the analyzed PE-specific genes varied over the course of biofilm succession. Some of the involved pathways were overexpressed only at specific successional stages, which we verified in detail via DESEQ2 analysis of differential abundance (Supplementary Table 3). In general, we detected a later onset of PE-specific pathways in the forest soil than in the same genes in the plastic soil biofilm communities (Fig. 5A and B).

During early colonization of PE foils in plastic soil (days 2–7), we detected high activity of genes involved in fatty acid degradation, whereas in forest soil, this activity occurred later during the midsuccessional stage (day 14). For example, transcripts of acyl-CoA dehydrogenase (*ACADM*), a putative acyltransferase (*atoB*), 3-ketoacyl-CoA thiolase (*fadA*) and fatty acid oxidation complex subunit alpha (*fadJ*) were expressed and highly abundant in this initial biofilm stage on PE foil in plastic soil (Fig. 5A). All of these genes are involved in either aerobic long-chain fatty acid degradation (*fadB* and *fadA*) or the anaerobic equivalent of the same pathway (*fadJ*). Even though transcripts of *atoB* and *fadA*  were detected in one glass sample on day 2, fatty acid degradation was less prevalent on glass than on PE foil in plastic soil for the remaining biofilm stages (Fig. 5A).

The initial biofilm stage on day 2 was also shaped by genes encoding membrane transporters such as the ATP-binding proteins (ABC transporters) LivF, LivH, LivM and LivG, whose transcripts were upregulated in both the glass and PE biofilm communities in the forest and plastic soils (Fig. 5A and B). The transcripts of the long-chain fatty acid transporter FadL and the TRAP-like transport protein Yiao, both involved in the transport of aliphatic hydrocarbons, were active in two initial biofilm samples on PE foil in plastic soil during days 2 and 7, as well as in a mature PE-associated biofilm sample on day 53 (Fig. 5A). In forest soil, these two transporters were active only in the biofilm samples collected on days 7–14 (Fig. 5B).

The genes involved in the degradation of n-alkanes included alkane monooxygenase (*alkM*), S-(hydroxymethyl)glutathione dehydrogenase (*frmA*), aldehyde dehydrogenase (*ALDH*) and long-chain alkane monooxygenase (*ladA*), all of which are also active during the early successional stages (days 2–7) of PE-associated biofilms from plastic soil (Fig. 5A) and slightly later (days 7–14) in biofilm communities from forest soil (Fig. 5B). The expression of genes related to n-alkane degradation was therefore highest in the early- to mid-successional stages and decreased in activity toward the late biofilm communities. Other enriched pathways included genes involved in the breakdown of secondary plant metabolites and natural terpenes, such as geraniol and citronellol (*atuC, atuD* and *atuF*), all of which were active during days 7–14 in forest soil and during the first sampling point (day 2) in plastic soil (Fig. 5). The only exception was the related isohexenylglutaconyl-CoA hydratase (*atuE*), which was active exclusively during the late successional stage of day 53 in both soils (Fig. 5).

The mid-successional stage in forest soil is characterized by the expression of transcripts encoding enzymes involved in fatty acid beta-degradation, such as FadJ (3-hydroxyacyl-CoA dehydrogenase), FadB (fatty acid oxidation complex subunit alpha) and ACADM (acyl-CoA dehydrogenase), whose expression of transcripts peaks on day 14 (Fig. 5). Additionally, genes involved in the pathways of geraniol degradation were overexpressed during this phase of biofilm development. Notably, in the context of possible PE-degrading pathways, the extracellular lipase gene *lip*, as well as the gene encoding acyl-CoA dehydrogenase *DCAA*, which is capable of degrading caprolactam, the monomer of nylon 6, was overexpressed. Moreover, we found that almost all the genes involved in the N-alkane degradation pathway were overly active at certain biofilm stages (Fig. 5). Similar to genes expressed in plastic soil, the alkane 1 monooxygenase *alkM* (candidate for PE degradation) was active during day 7 and day 14 of biofilm formation and remained downregulated during both the initial biofilm phase (day 2) and late biofilm succession (day 53). To our surprise, we found that transcripts encoding the polyethylene terephthalate-degrading enzyme PETase were present and expressed by the biofilm community in the early- and midsuccessional stages in forest soil (days 7 and 14) but were entirely absent in plastic soil.

Overall, the late biofilm samples (day 53) presented only a few genes whose expression was above average (Fig. 5). Except for a single PE sample in plastic soil (Fig. 5A, PE1 day 53), we observed an overall decline in the expression of genes relevant for PE degradation toward the late-successional phases of biofilm growth (Fig. 6). In fact, most genes that were highly active in the preceding biofilm stages were underexpressed in the mature biofilm communities. Among the genes involved in fatty acid beta-oxidation, only *fadB* and *fadN* (both fatty acid oxidation complex subunit alpha) were slightly above the average in the late biofilm stage of plastic soil and forest soil, respectively (Figs. 5 and 6). Other earlier abundant enzymes, such as ABC transporter genes or the PE-specific candidate genes Alkane 1 monooxygenase *alkM* and the extracellular lipase *lip*, were markedly underrepresented in the late biofilm stage. Moreover, nonspecific genes involved in the biosynthesis of secondary metabolites (3-oxoacyl-[acyl-carrier protein] reductase *fabG* and a putative enoyl-CoA hydratase *atuE*) were overrepresented on day 53 of incubation in both soils. One sample of the biofilm community on PE foil (PE1day53) presented remarkably high expression of PE-specific genes compared with the other samples from the same sampling day (Fig. 5A). These highly expressed genes included ABC transporter genes (e.g., *livH, livF* and *livG/cysA*), as well as genes involved in fatty acid metabolism, including the extracellular *lip* lipase (lonizing lipase precursor) and *fadA* (3-ketoacyl-CoA thiolase). In the forest soil, we observed a clear functional pattern that distinguished the different successional biofilm stages from each other with respect to PE-specific genes. (Fig. 5B). In particular, the genes encoding dehydrogenases, specifically *fadE* (which encodes the protein acyl-CoA dehydrogenase) and *frmA* (which encodes the protein S-(hydroxymethyl)glutathione dehydrogenase), showed considerably higher activity than in the later biofilm stages. Unique for the sequenced PE biofilm communities in this early stage was the overexpression of the genes *phaZ*, which encodes the extracellularly secreted carboxylic-ester hydrolase (Esterase PHB depolymerase), in the PE communities compared with the biofilm on glass (Fig. 5).

### The plastic soil microbial community highly expresses *nif* genes to overcome nitrogen limitation caused by PEs in mature biofilms

In addition to genes related to PE degradation, we detected the expression of genes responsible for microbial nitrogen fixation (Fig. 7) in the biofilm community. Most striking was the increase in the expression of a number of *nif* genes, which encode a variety of nitrogenases and constitute an important part of the microbial nitrogen fixation pathway, over the course of biofilm succession in plastic soil (Fig. 7A).

In plastic soil, the expression of *nifD*, *nifH*, *nifK*, *nifN* and *nifT* was the highest in the mature biofilm stage, with *nifH* reaching the highest gene expression on PE foil after 53 days of incubation (Fig. 7A). However, transcripts of *nifD*, *nifH* and *nifK* were present during the mid-successional biofilm stage on two PE samples (Fig. 7) and on a single glass sample on day 2 of incubation. The highest expression levels were detected in the PE samples at this stage of biofilm succession, even though the biofilms of a single glass sample also expressed four of the *nif* genes at low abundances. To our surprise, we could not detect a single transcript of a *nif* gene related to nitrogen fixation in our forest soil biofilm community.

### Biofilm succession and surface coverage via SEM image analysis

**Fig. 8:**
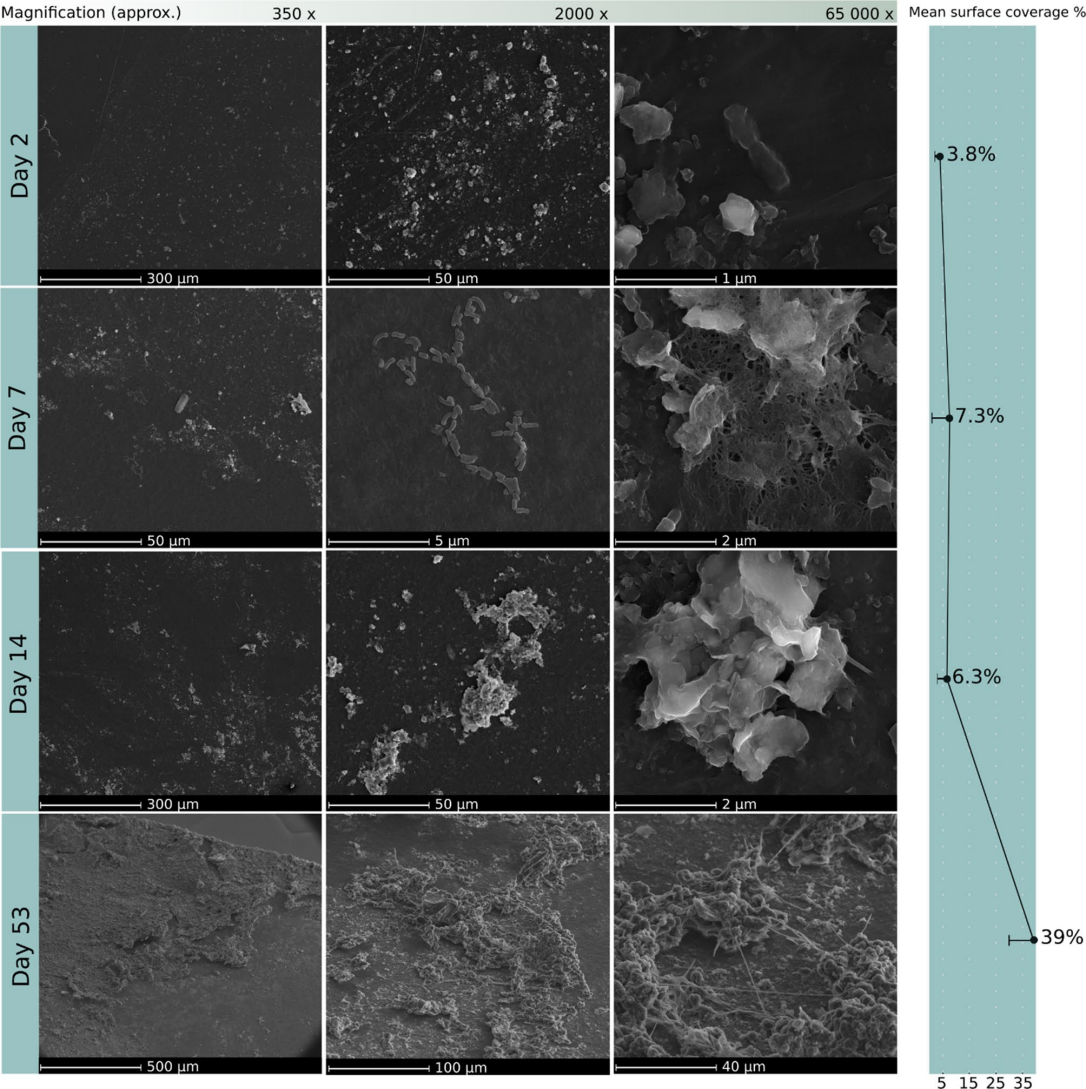
Plastic soil: SEM image analysis and biofilm coverage on PE foil in plastic soil during 53 days of incubation. The mean surface coverage was calculated at 200x magnification (*n* = 5). We detected a strong increase in biofilm coverage over time on the PE surface in the plastic soil

**Fig. 9:**
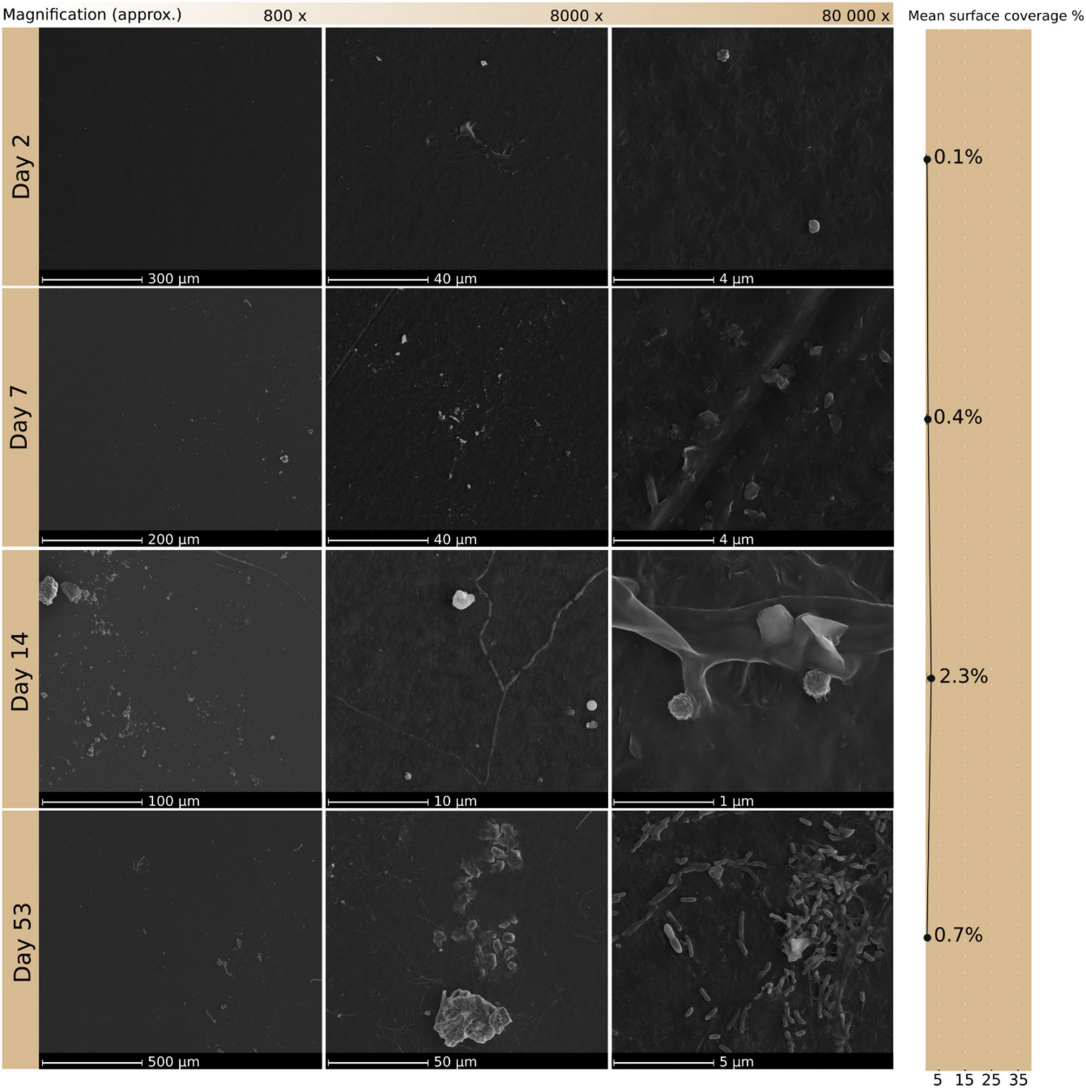
Forest soil: SEM image analysis and biofilm coverage on PE foil in forest soil during 53 days of incubation. The mean surface coverage was calculated at 200x magnification (*n* = 5). Biofilm coverage did not increase significantly over time on the PE surface in forest soil

In addition to the transcriptomic data of the microbial biofilm communities on soil-buried PE foil, we used SEM image analysis to visually follow the growth and successional development of the biofilm stages in both soils (Figs. 8 and 9). To quantify the surface coverage, we used threshold-based image analysis, which depicted biofilm structures from the background surface, and calculated the extent of microbial coverage (Supplementary Figure S3).

Initial biofilm growth was visible on day 2 of incubation in both soil types. Over time, we observed greater surface coverage of biofilms in plastic soil (Fig. 8) than in forest soil (Fig. 9). The biofilm complexity on the PE surface increased with increasing incubation time in both soils, with a variety of morphological biofilm features. Structures of extracellular polymeric substances (EPSs) were detected within the biofilm, where strings connected the thick microbial mats to the surface (Fig. 8: day 53).

We found various shapes of bacterial cells (e.g., singular cocci and rod-shaped bacteria as well as streptococci and tetrads) in close arrangements on the surface and embedded within surface grooves of the PE foil (Figs. 8 and 9). Most apparent were the large fungal hyphal structures from day 14 on and several fungal spores on the PE foil. In the later biofilm stage within the plastic soil, we detected an increased number of filamentous bacteria, as well as Streptococci and chains of Bacilli (Fig. 7: day 53). In general, the surface coverage of PE samples with biofilms in plastic soil was greater than that of those incubated in forest soil. The PE surface in the plastic soil was covered with thick biofilms after 53 days of incubation, where the mean coverage reached 39% of the PE surface (Fig. 8). In particular, in the middle stages of biofilm growth (14 days), numerous bacterial cell aggregates were visible on the PE surface, as were fungal structures such as hyphae and spores, which formed a substantial microbial layer on top of the surface. In forest soil, the mean surface coverage was highest on day 14 at 2.3% (Fig. 9: day 14) and did not reach higher coverages throughout the incubation. Considering these low coverage numbers, we detected an earlier onset of biofilm development in plastic soil than in forest soil, similar to the patterns visible in the metatranscriptome data.

## Discussion

### Time-dependent changes in the microbial biofilm community on PE surfaces

In both soils, we found a time-dependent change in the taxonomic composition of the microbial community and their PE-specific gene expression pattern despite the origin of the colonizing microbial community (plastic soil vs. forest soil). Notably, compared with their initial stages, the sequenced PE-associated biofilm communities became less diverse over time. This finding indicates that the successional age of the biofilm has a stronger effect on the microbial community composition than does the original soil community, which aligns with observations from other biofilm studies on polymer surfaces [36, 38, 53–55].

In general, biofilm succession refers to the gradual change in the composition and structure of a microbial biofilm community over time. In our case, the most significant shifts occurred between days 14 and 53 of incubation, during which the microbial biofilm communities in both soil types became less diverse and distinct from their earlier stages. Similar patterns were observed in soil bacterial biofilms on low-density polyethylene (LDPE), where significant successional changes occurred only after extended incubation periods [56]. Other studies have reported time-dependent dynamics in microbial biofilms on marine plastic debris, noting that biofilms mature and become independent of the surface characteristics of the material after approximately two weeks [57]. Our data support these findings, as we observed that most transcripts of plastic-associated taxa were prevalent within the first two sampling points (Days 2 and 7). The observed decrease in microbial diversity on soil-buried plastic debris at the microscale (both in terms of time and scale) may explain the frequently reported reduction in microbial diversity within the soil plastisphere [10, 58–60].

In plastic-contaminated soil, taxa known for plastic degradation, such as *Aspergillus*, *Rhodococcus*, *Bacillus*, *Nocardioides*, *Streptomyces*, and *Nocardia*, were present in the early successional stages of the PE biofilm but decreased in abundance as the biofilm matured. This suggests that although initial colonization of plastic debris might occur more rapidly in aquatic environments, the time required for plastic degradation and adaptation to the physico-chemical properties of polymers is also a significant ecological driver in terrestrial systems [56, 57].

### Ecological drivers of biofilm succession

During the process of biofilm succession, various selective pressures shape the community composition. Initially, bottom-up selection, driven by the availability of heterotrophic resources such as plastic-derived carbon, plays a crucial role. However, as biofilms mature, both bottom-up and top-down mechanisms, such as competition among higher trophic levels, become important in regulating the community [61].

Previous research on plastisphere communities, particularly in aquatic environments, has traditionally focused on heterotrophic bacteria. However, studies have increasingly reported the rise of opportunistic cross-feeders, grazers, and phototrophs during biofilm maturation [41, 57, 62]. According to our observations and those of others, heterotrophic plastic-degrading microorganisms often occur at low abundances, making it difficult for them to compete with the dominant autotrophs in mature biofilms [38, 63]. As the biofilm thickens, contact with the plastic surface diminishes, reducing the potential for direct microbial degradation of the polymer. We conclude that once biofilm succession reaches a mature stage (days 14–53), the dominance of (photo)autotrophs significantly hampers efficient plastic degradation due to top-down selection pressures. Our research indicates that these dynamics are not only relevant to marine plastic debris but also apply to dynamics within the soil plastisphere [56]. In our study, after initial enrichment of plastic-associated taxa during the early stages, the biofilm community shifted toward dominance of phototrophic Cyanobacteria and green algae (*Chlorophycae* and *Trebouxiophyceae*), alongside biofilm grazers such as *Acanthamoeba* and *Vermamoeba*. This shift likely reflects a depletion of easily accessible plastic-derived organic matter, leading to autotrophic taxa and predators becoming more prevalent as a clear example of top-down selection [38, 41, 57, 61–63].

Unlike in aquatic systems, our data suggest that biofilm maturation in soil leads to the development of a community resembling natural soil crusts. These well-described model ecosystems consist of microbial filaments, aggregates, Cyanobacteria, and algae embedded in an organic matrix [64, 65]. Owing to their extreme tolerance to desiccation and nutrient deficiencies and their high photosynthetic activity, they can accumulate organic matter and build a microhabitat independent of their original surface properties [66, 67]. While the biodegrading potential of Cyanobacteria and Algae might have been largely overlooked for a long period, some algal species (*S. dimorphus*,* A. spiroides*,* P. lucidum*, *O. subbrevis*, and *N. pupula*) were recently found to possess PE-degrading features [68–71]. However, it is important to consider the availability of light for photosynthesis when studying microbial responses to plastic debris in soil, particularly for biofilms attached to debris in the upper soil layers. We speculate that in deeper soil layers, where light is unavailable, other selection mechanisms might prevail, potentially allowing plastic-degrading taxa more time to act on the plastic surface. A detailed analysis of plastic-attached microbial communities in soil while also considering the depth of the soil layer might help to further understand the different strategies in the terrestrial plastisphere.

### Enhanced colonization efficiency of microbial communities from plastic-contaminated soil

Our predictions that soil types and their native microbial communities differ in their colonization potential and PE specificity were confirmed by our observations. Scanning electron microscopy (SEM) analysis revealed more rapid colonization of the PE foil by microorganisms from the plastic-contaminated soil community, as evidenced by greater biofilm coverage and less variation in biofilm-associated genes. Recent mining of environmental metagenomes revealed a correlation between the level of pollution in a given ecosystem and the abundance of plastic-degrading enzymes, suggesting that areas of high pollution are already driving microbiomes to evolve the potential for plastic degradation [72]. Following these findings, we assume that exposure to plastic debris shaped our studied microbial community in plastic soil toward a community of highly efficient colonizers that are able to attach to the PE surface quite rapidly. As soil matrices consist of numerous available surfaces on which biofilms can be established (e.g., soil particles and biotic structures), the competition for “attachment” might be less severe, yet biofilm-associated bacteria in soil were shown to have fitness advantages compared with planktonic organisms [73].

In highly plastic-polluted soils, competition for rapid biofilm succession might be greater than that in natural soils because of the scarcity of available substrates and the selective pressure to establish a functional biofilm community. Research has shown that nitrogen fixation is the rate-limiting step of microbial polymer degradation in soil, which can be overcome by the establishment of a functioning N-uptake system provided by phototrophs and other N-fixing microorganisms [74, 75]. In our study, we observed an increase in N-fixing phototrophs in the late stages of biofilm succession in the plastic soil but not in the forest soil. This increase in the cyanobacterial genera *Nostoc*,* Desmonostoc*, and *Tolypothrix* was accompanied by an increase in genes responsible for N fixation, such as *nifD*, *nifH*,* nifK*,* nifN* and *nifT*, all of which are absent in the natural forest soil community.

Notably, the impact of plastic debris on the prevalence of these nitrogen fixation indicator genes in the soil is a rather new emerging area of research, with contrasting findings thus far. Regarding LDPE, Feng and colleagues reported that low doses of LDPE debris in soil did not significantly affect the abundance of marker genes (*nifD*, *nifH*, and *nifK*) related to nitrogen fixation stages [76]. However, in another study by Rong and colleagues, both low and high doses of LDPE led to increased gene abundance of these genes, which was due to an increase in genera related to nitrogen fixation [77]. This finding is also supported by our observation that the N-fixing bacterial taxa *Gemmataceae* and *Gemmata* followed the same trend and increased in abundance during the late biofilm stage (days 14–53). Earlier research revealed that cyanobacteria and other N-fixing microorganisms facilitate polymer degradation by providing missing nitrogen for other hydrocarbon-degrading species [75, 78]. To decompose N-poor substrates such as plastic, microorganisms need to establish a nitrogen uptake system from the surrounding matrix [79]. This means that taxa capable of directly utilizing plastic-derived carbon and taxa that carry out important secondary functions within the interactive network could thrive in the system. Our results contribute to this novel area of research and suggest that one distinct feature of the terrestrial plastisphere is the enrichment of marker genes involved in nitrogen fixation. In other words, thriving autotrophic microbes exhibit a competitive advantage within this terrestrial plastisphere habitat, which is eventually reflected in their genetic potential to fix inorganic nitrogen [72, 73, 75, 78].

### Detection of PE-degrading enzymes

An earlier metagenomic study of plastic debris in water concluded that the surface of plastic debris is enriched with taxa that possess discrete sets of genes compared with those in the surrounding bulk waters [21]. Extending this notion to the terrestrial realm, we expected that the buried PE foil would similarly enrich for PE-specific genes, especially compared with glass slides or the surrounding bulk soils. Therefore, we predicted that the microbial community in plastic-contaminated soil would exhibit a broader array of genes related to PE degradation. In their 2021 study, Zrimec and colleagues reported that a high abundance of plastic-degrading microbial enzymes was correlated with a relatively high degree of plastic pollution in a system [72].

In terms of potentially PE-degrading enzymes, our analysis revealed that the alkane monooxygenase gene, *alkB1_2/alkM*, was upregulated during the early stages of biofilm succession in plastic-contaminated soil. This upregulation suggests active hydroxylation of polymer-derived compounds during the initial phases of biofilm formation. This finding aligns with the presence of plastic-degrading taxa predominantly during the early successional stages, although the specific taxonomic origin of *alkB* and its encoding genes remains unidentified. Additionally, we tested other known PE-degrading enzymes, such as the laccase gene from *Rhodococcus ruber* and manganese peroxidase (*mpn*) lignin-degrading fungi such as *Phanerochaete chrysosporium* and *Trametes versicolor* [80, 81]. Although *Rhodococcus* was abundant in the early stages, we did not detect laccase transcripts in our metatranscriptome.

As PE degradation progresses, dedicated transport systems may facilitate the uptake of short-chain PE fragments posthydroxylation [82, 83]. Our data support this, as we observed the presence of various transcripts of membrane transporters, such as the long-chain fatty acid transporter (FadL) and ABC transporters, which play crucial roles in the uptake of hydroxylated hydrocarbons. The high expression of these transporter genes in our PE-associated biofilm suggests an active mechanism for importing plastic-derived oligomers from the PE surface, which is consistent with previous studies.

The long-chain fatty acid transporter (FadL) is known to play a major role in alkane transport and is present in bacteria involved in the biodegradation of xenobiotics [84]. In addition, Zadjelovic and colleagues showed in their extensive metaproteome study that both TRAP-like membrane transporters and ATP-binding proteins (ABC transporters) were upregulated in the presence of weathered PE [25]. Moreover, the actinomycete *Rhodococcus rhodochrous* was reported to assimilate PE oligomers 600 Da in size through cassettes of ATP-binding proteins [85]. Our detection of high expression of transporter genes (ABC transporters) in our PE-associated microbial biofilm is consistent with these findings, which suggests the active uptake of plastic-derived oligomers from the PE surface.

Our metatranscriptomic data revealed that during the early successional stages, plastic-degrading taxa were present along with the upregulation of plastic-degrading enzymes. We observed that transcripts of the PE-degrading enzymes AlkB1/AlkM and transcripts encoding transporters such as FadL, LivG, LivF, LivH and LivM and the fatty acid β-oxidation pathway were active during the maturation of the biofilm. To our surprise, the number of PE-related genes present and expressed in the natural forest soil biofilm community was very similar to the number of genes expressed in the highly polluted plastic soil community, in contrast to earlier predictions. Moreover, the transcriptional response of the PE-attached biofilm community followed a clear successional pattern over time, with distinct phases in which only certain PE-specific pathways were active. Overall, the presence and expression of these enzymes suggest that the microbial community in plastic soil has the potential to degrade plastic compounds, although their expression patterns over time also suggest that other metabolic dynamics may be involved in shaping the microbial community in biofilms. We can further conclude from our findings that even pristine forest soils, which lack major exposure to plastic debris, still harbor a variety of plastic-associated genes (*PETase, alkB*, etc.) and therefore carry the enzymatic potential for microbial plastic degradation. In the quest for plastic-degrading enzymes, these findings might shift the attention from highly disturbed ecosystems (e.g., landfills) toward natural and diverse soil ecosystems where PE-specific enzymes seem to be present.

## Conclusion

In conclusion, our metatranscriptome study provides new insights into the taxonomic and functional dynamics of microbial biofilm succession in the terrestrial plastisphere. We observed a time-dependent shift in the composition of the biofilm community, with plastic-degrading taxa being present in the early successional stage and phototrophic organisms and grazers dominating the later stages. Here, we observed a soil crust-like biofilm community as the most mature successional stage on PE in soil, most likely resulting from top-down selection pressure rather than actual plastic degradation once nitrogen fixation by (photo)autotrophs became the limiting step. These observations also highlight the importance of considering the availability of light as a factor that influences microbial strategies for plastic degradation in soil. Further research on plastic-attached microbial communities in soil, considering the depth of the polluted soil layer, is needed to better understand the different physiological strategies of such systems. Our results also imply that even pristine forest soils without prior exposure to plastic debris harbor and express a variety of plastic-associated genes (*PETase, alkB*, etc.). However, despite the apparent metabolic potential of such natural soil communities, microbiomes that were previously exposed to plastic debris seem to be primed toward the colonization of PE and, most likely, PE degradation. Furthermore, we observed an increase in genes related to nitrogen fixation in the plastic soil community but not in the forest soil community. In this context, we demonstrated that the high abundance of nitrogen fixation genes is another essential metabolic adaptation of biofilm communities in highly plastic-polluted soil environments. Overall, these findings highlight the complex and dynamic nature of plastic-associated biofilms and suggest that different stages of biofilm development may be associated with different microbial functions and the potential for plastic degradation. This understanding is crucial in the ongoing quest for the discovery of plastic-degrading microorganisms and shows that whole-community dynamics should be considered for mitigating plastic pollution in soil ecosystems.

## Electronic supplementary material

Below is the link to the electronic supplementary material.

Supplementary Material 1

Supplementary Material 2

Supplementary Material 3

Supplementary Material 4

Supplementary Material 5

Supplementary Material 6

## Data Availability

All data generated or analysed during this study are included in this published article [and its supplementary information files]. Metagenomic and metatranscriptome raw data was uploaded to ENA under project accession number PRJEB60067 : https://www.ebi.ac.uk/ena/browser/view/PRJEB60067.
