# Supplementary figures and images for: Metatranscriptomics of microbial biofilm succession on HDPE foil: uncovering plastic-degrading potential in soil communities
Source: Environ Microbiome. 2024 Nov 21;19:95. doi: 10.1186/s40793-024-00621-1 (PMC11583400; doi:10.1186/s40793-024-00621-1)

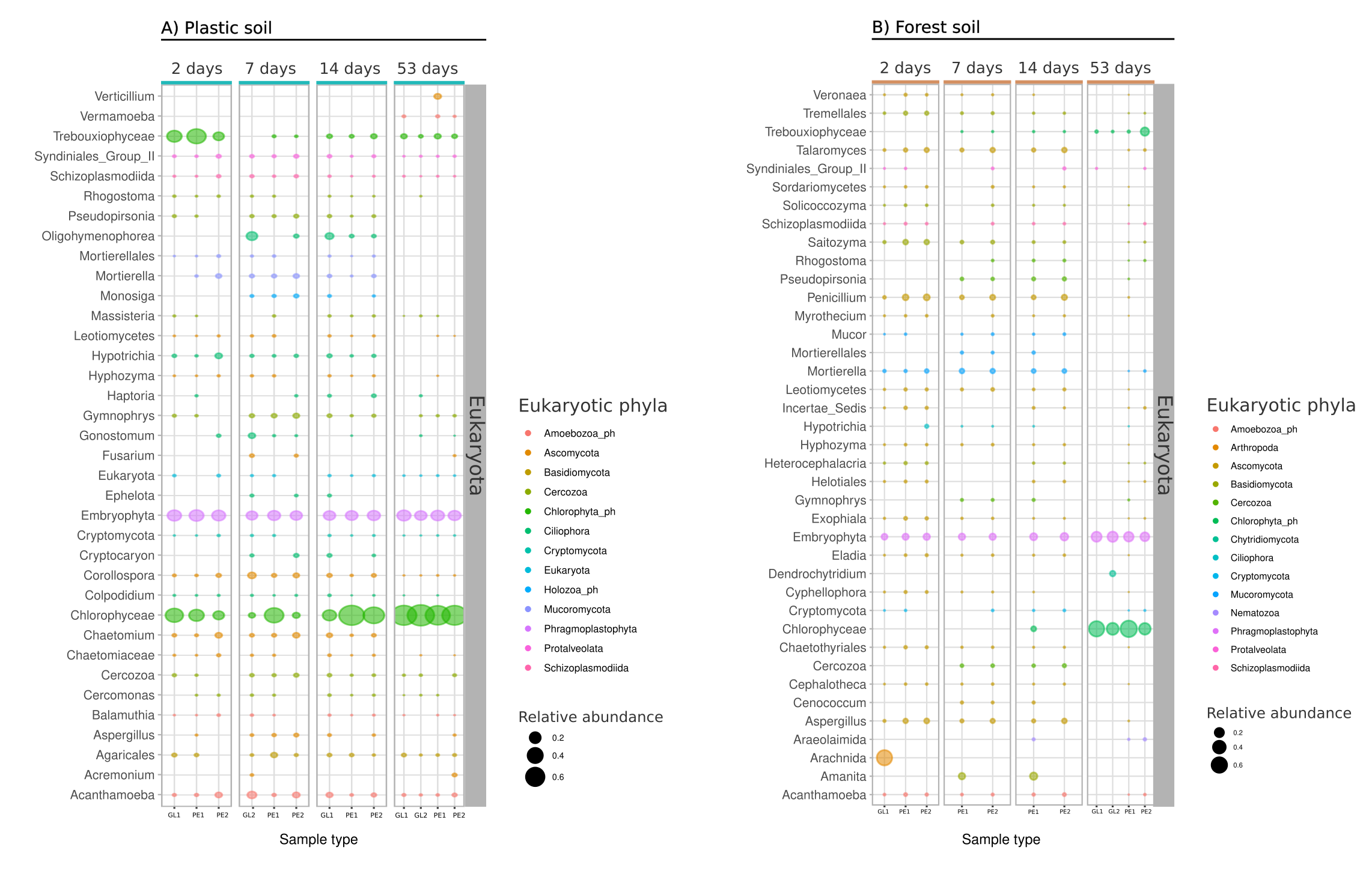

Supplement: Supplementary file 1 — Supplementary Material 1 [file 40793_2024_621_MOESM1_ESM.png]

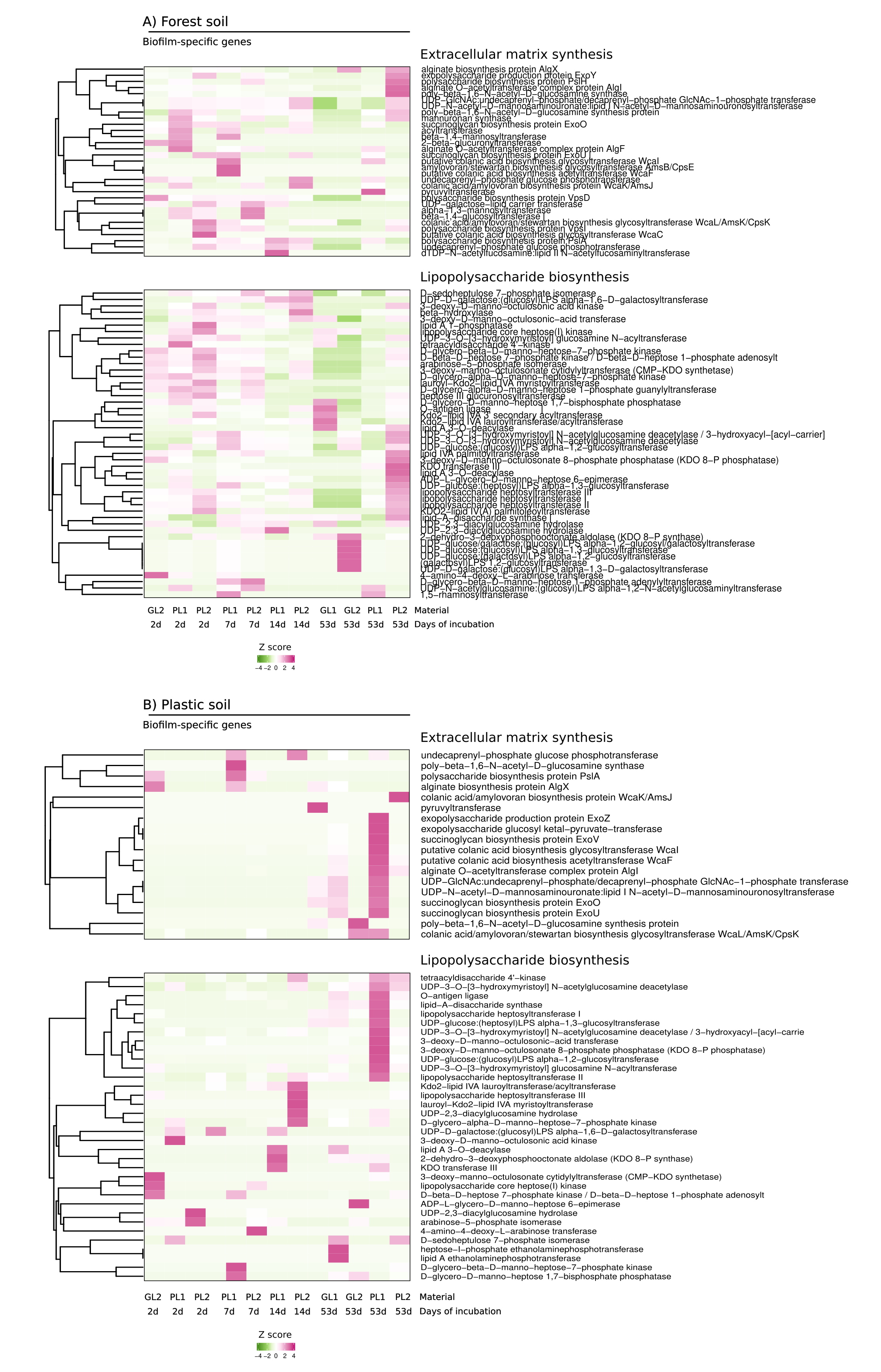

Supplement: Supplementary file 2 — Supplementary Material 2 [file 40793_2024_621_MOESM2_ESM.png]
