# Supplementary material for: Metatranscriptomics of microbial biofilm succession on HDPE foil: uncovering plastic-degrading potential in soil communities
Source: Environ Microbiome. 2024 Nov 21;19:95. doi: 10.1186/s40793-024-00621-1 (PMC11583400; doi:10.1186/s40793-024-00621-1)

Forest soil | Glass | Biofilm surface coverage (S_c)

2 days

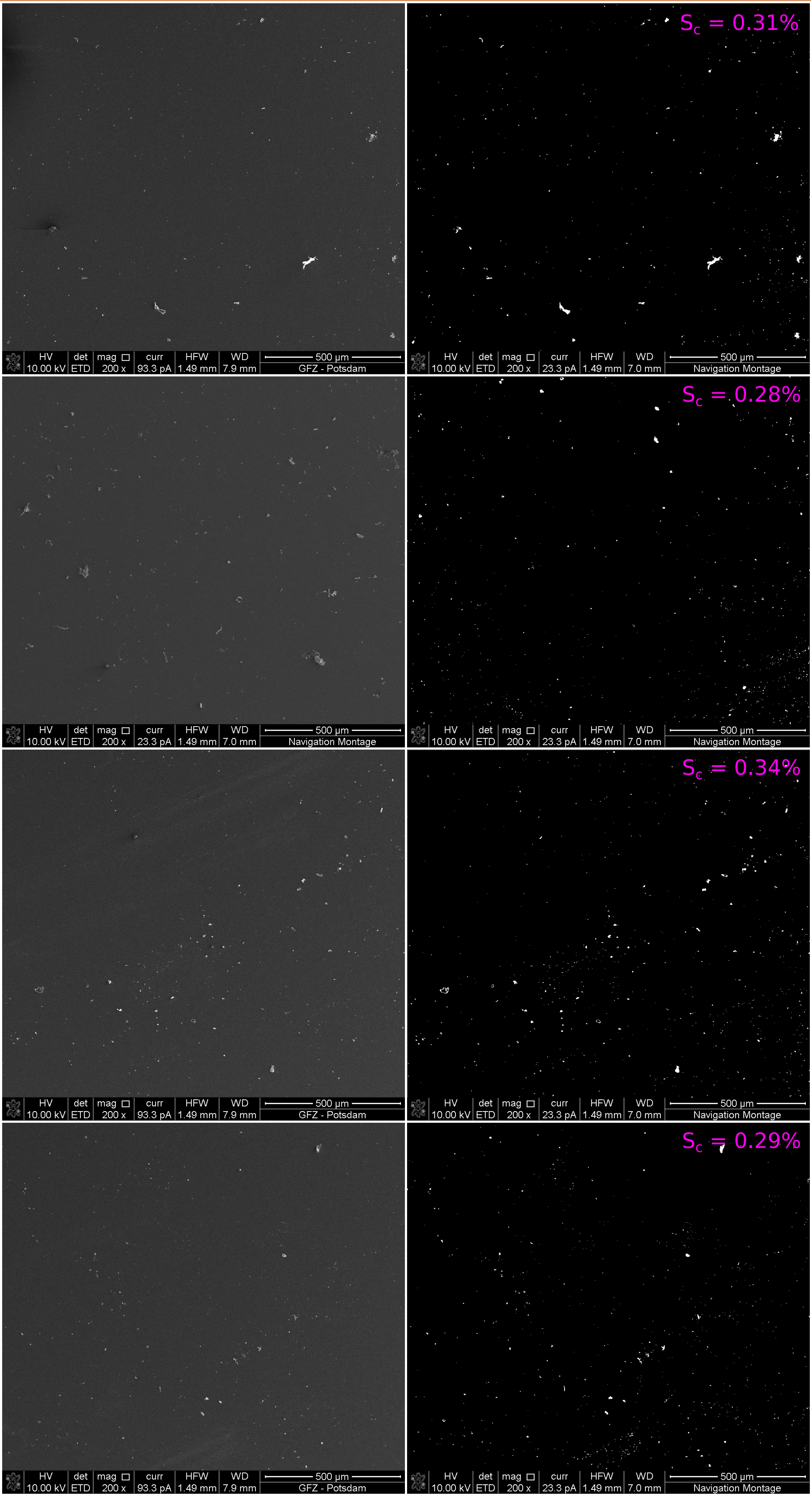

Forest soil | PE | Biofilm surface coverage (S_c)

2 days

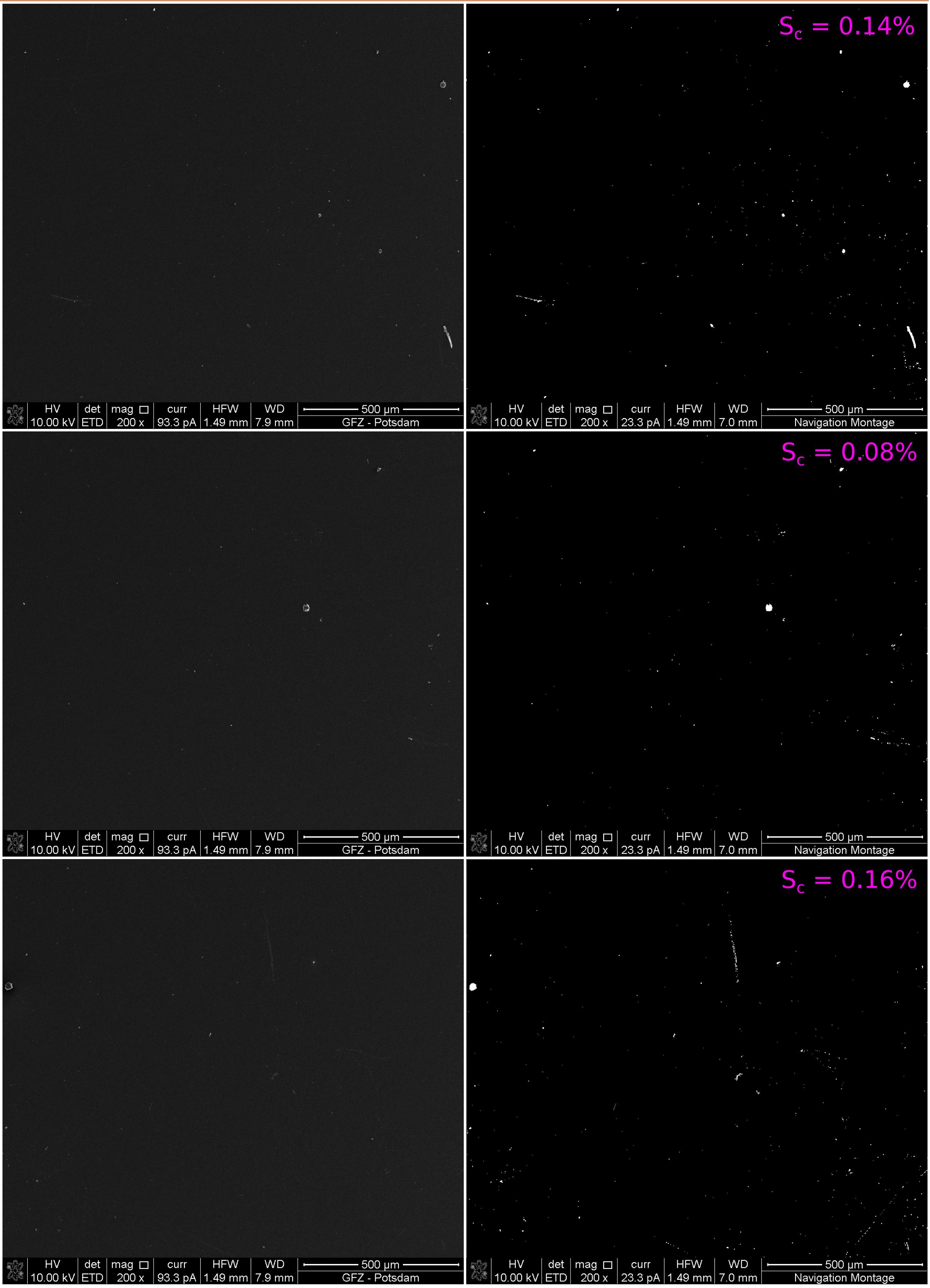

Plastic soil	Glass	Biofilm surface coverage (S_c)

2 days

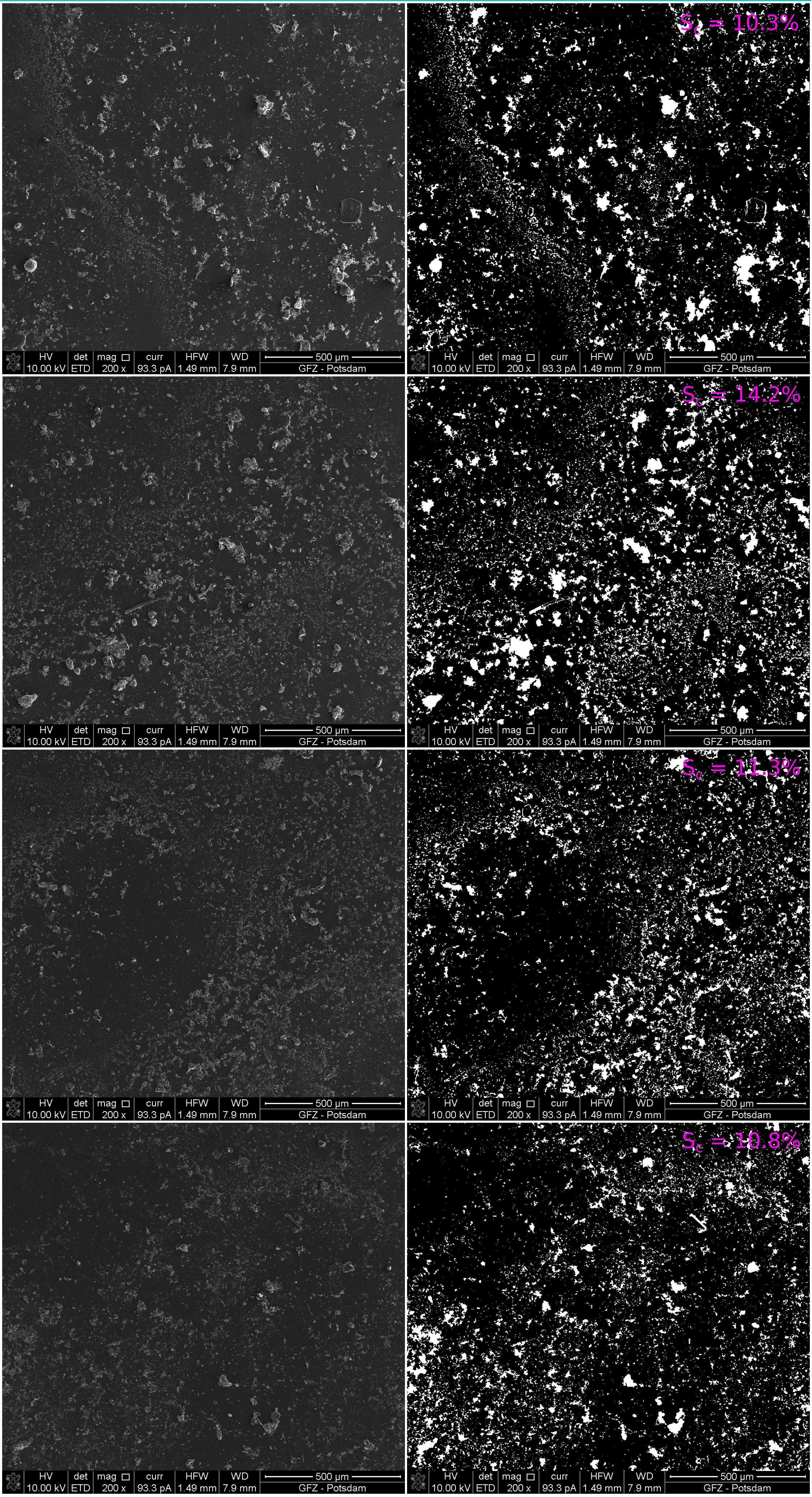

Plastic soil	PE	Biofilm surface coverage (S_c)

2 days

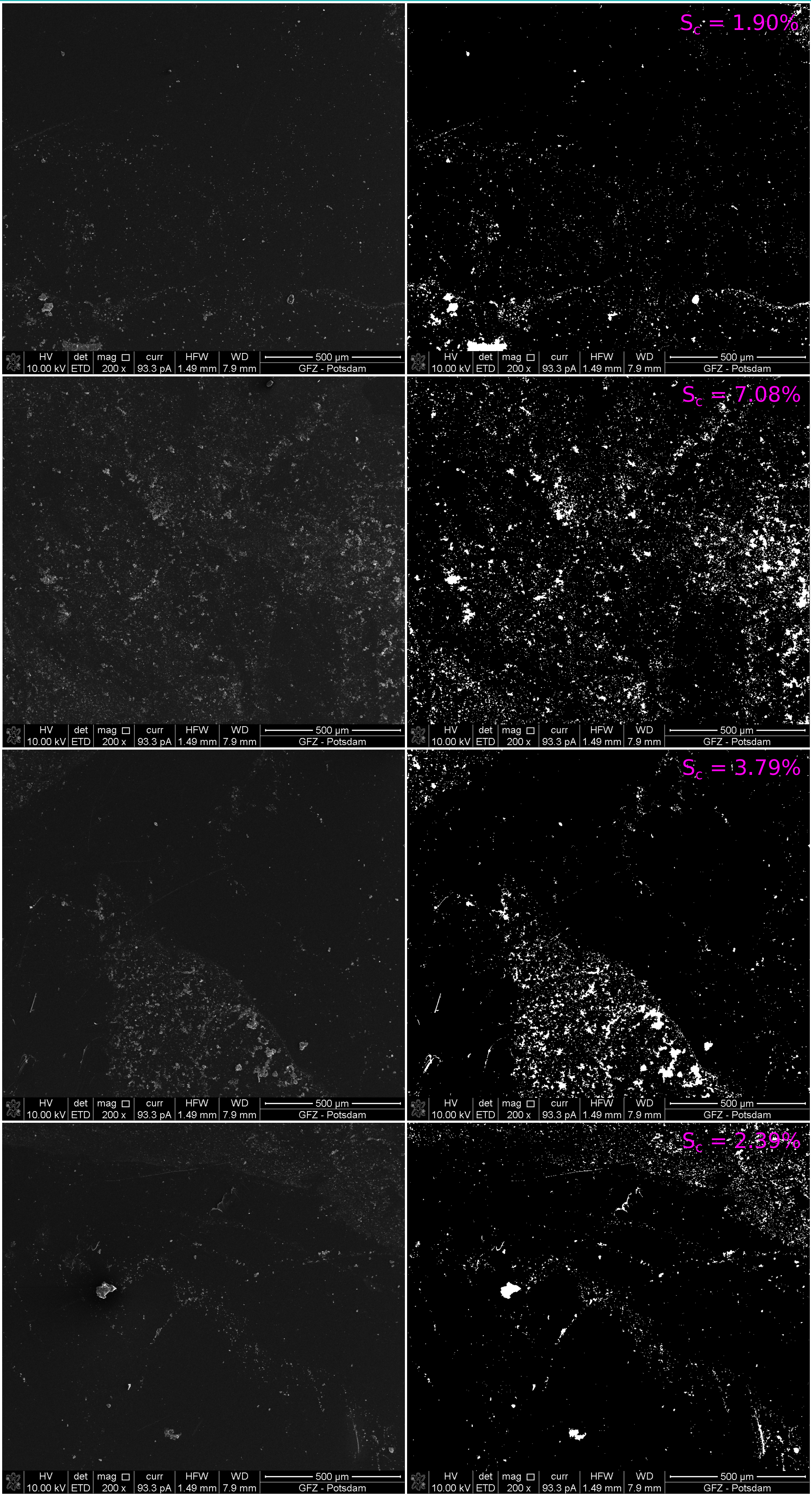

Forest soil

53 days

Glass

Biofilm surface coverage (S_c)

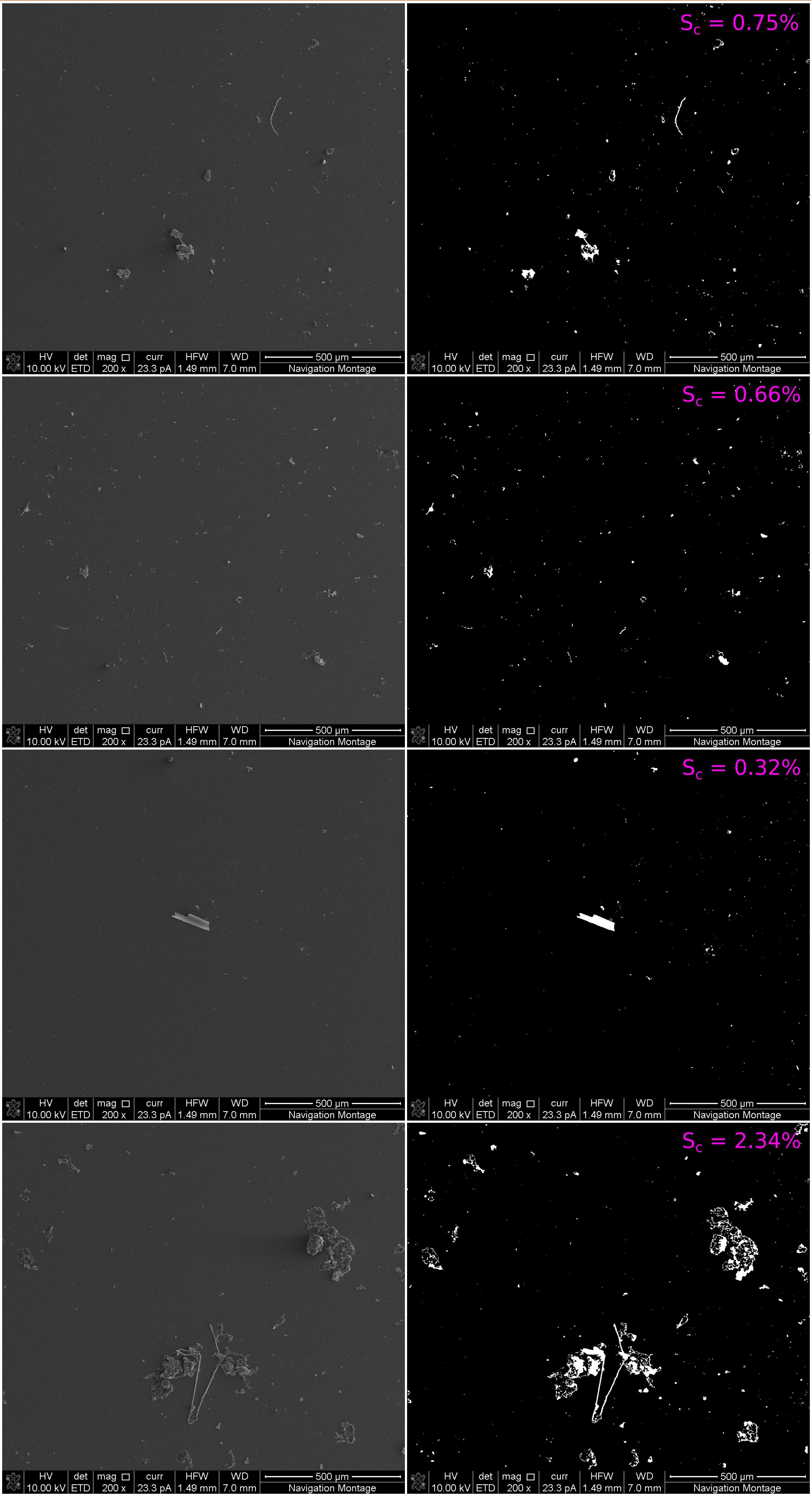

Forest soil | PE | Biofilm surface coverage (S_c)

53 days

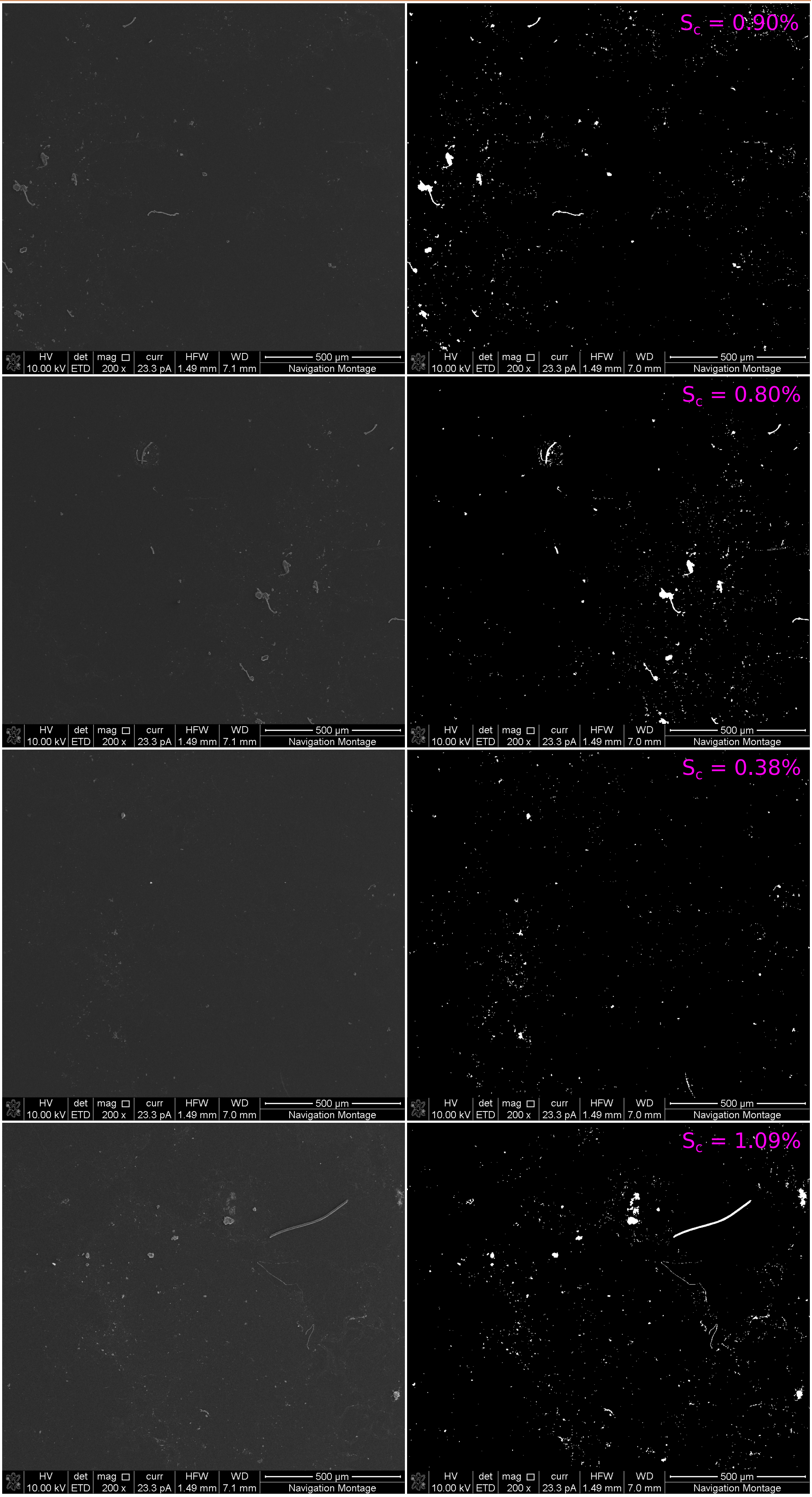

Plastic soil | Glass | Biofilm surface coverage (S_c)

53 days

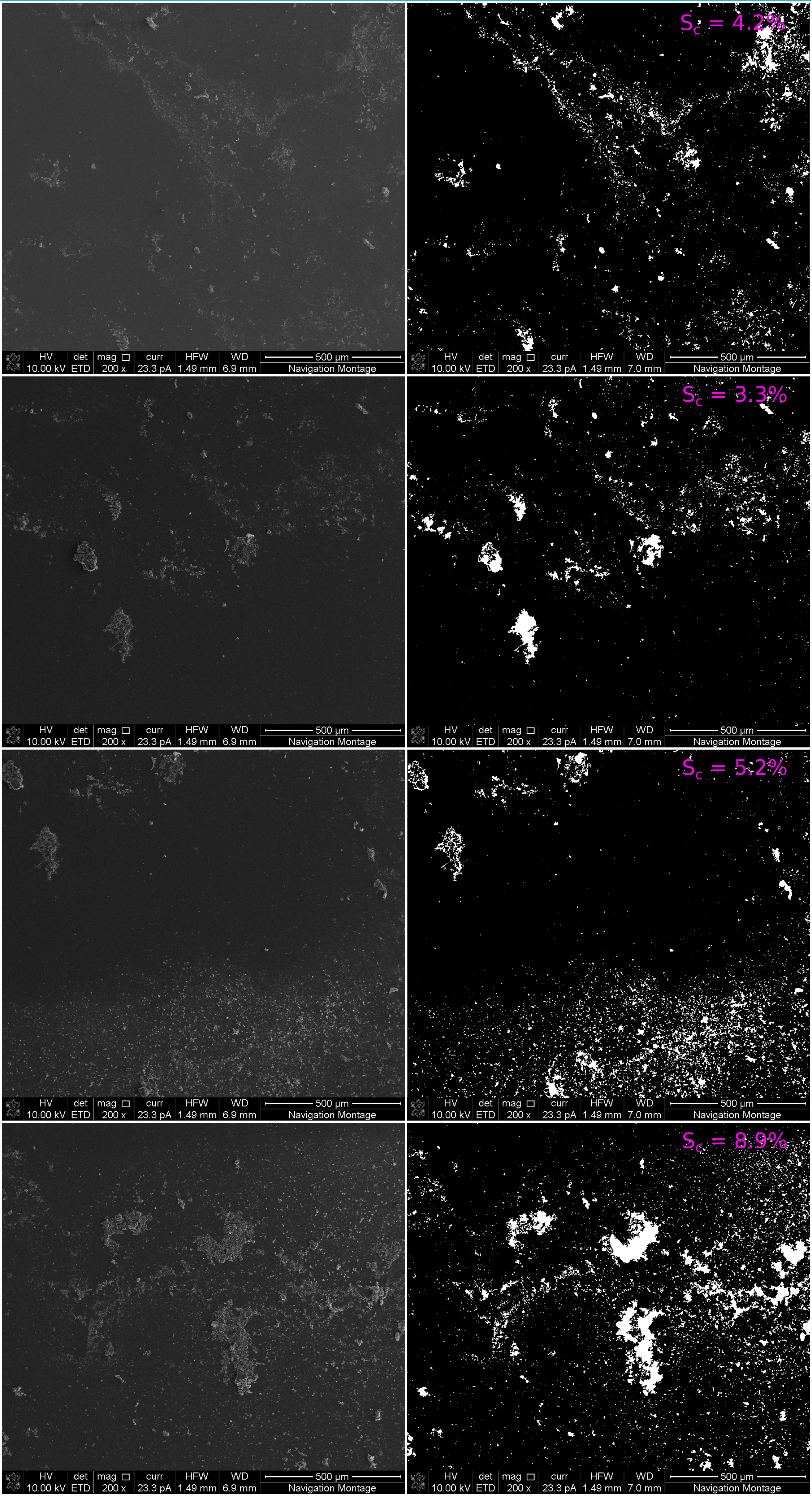

Plastic soil	PE	Biofilm surface coverage (S_c)

53 days

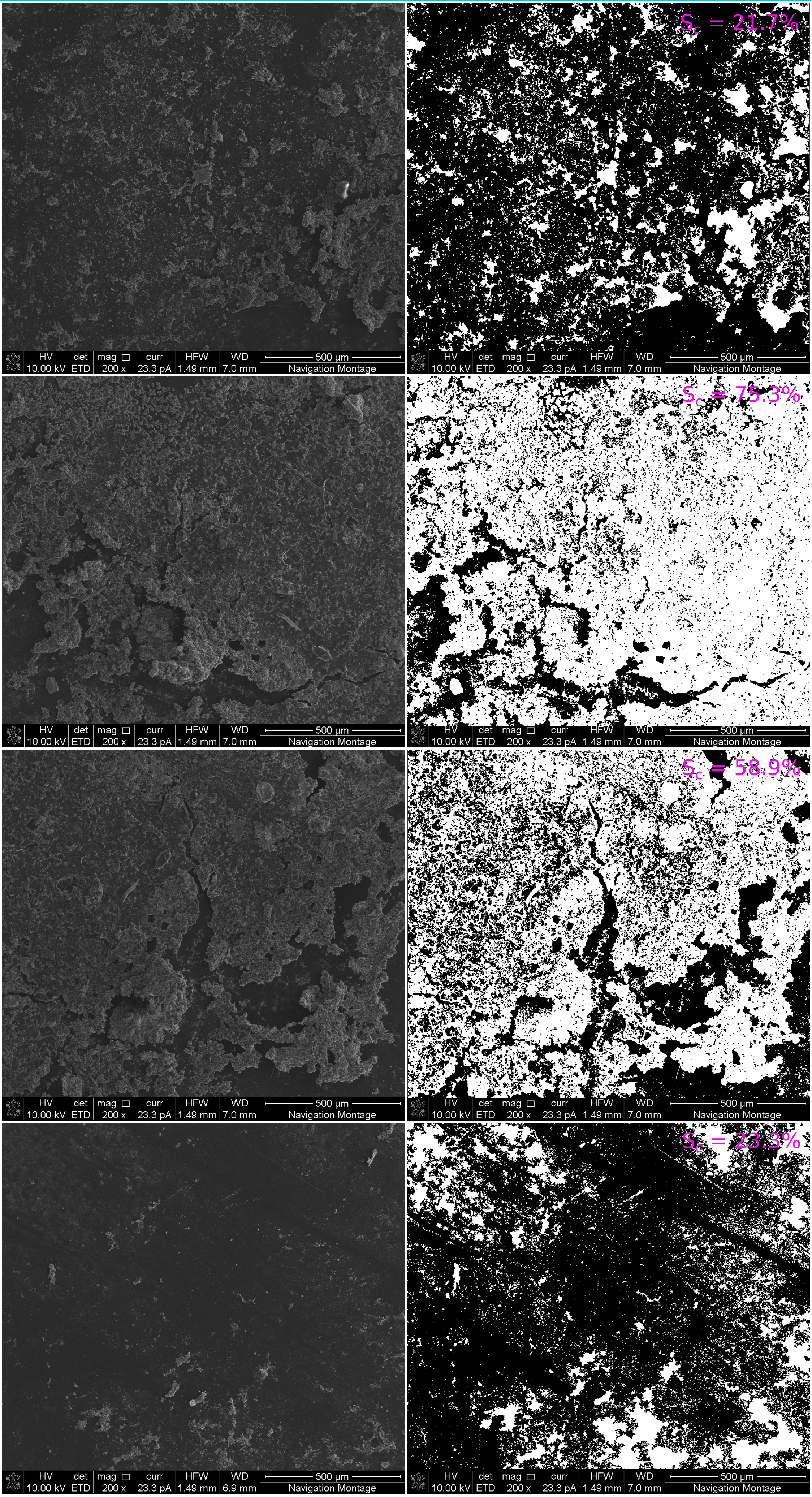

Supplement: Supplementary file 3 — Supplementary Material 3 [file 40793_2024_621_MOESM3_ESM.pdf]
